# LMW-E/CDK2 Deregulates Acinar Morphogenesis, Induces Tumorigenesis, and Associates with the Activated b-Raf-ERK1/2-mTOR Pathway in Breast Cancer Patients

**DOI:** 10.1371/journal.pgen.1002538

**Published:** 2012-03-29

**Authors:** MyLinh T. Duong, Said Akli, Caimiao Wei, Hannah F. Wingate, Wenbin Liu, Yiling Lu, Min Yi, Gordon B. Mills, Kelly K. Hunt, Khandan Keyomarsi

**Affiliations:** 1 Department of Experimental Radiation Oncology, The University of Texas MD Anderson Cancer Center, Houston, Texas, United States of America; 2 Graduate School of Biomedical Sciences, The University of Texas, Houston, Texas, United States of America; 3 Department of Biostatistics, The University of Texas MD Anderson Cancer Center, Houston, Texas, United States of America; 4 Department of Surgical Oncology, The University of Texas MD Anderson Cancer Center, Houston, Texas, United States of America; 5 Department of Bioinformatics and Computational Biology, The University of Texas MD Anderson Cancer Center, Houston, Texas, United States of America; 6 Department of Systems Biology, The University of Texas MD Anderson Cancer Center, Houston, Texas, United States of America; University of Washington, United States of America

## Abstract

Elastase-mediated cleavage of cyclin E generates low molecular weight cyclin E (LMW-E) isoforms exhibiting enhanced CDK2–associated kinase activity and resistance to inhibition by CDK inhibitors p21 and p27. Approximately 27% of breast cancers express high LMW-E protein levels, which significantly correlates with poor survival. The objective of this study was to identify the signaling pathway(s) deregulated by LMW-E expression in breast cancer patients and to identify pharmaceutical agents to effectively target this pathway. Ectopic LMW-E expression in nontumorigenic human mammary epithelial cells (hMECs) was sufficient to generate xenografts with greater tumorigenic potential than full-length cyclin E, and the tumorigenicity was augmented by *in vivo* passaging. However, cyclin E mutants unable to interact with CDK2 protected hMECs from tumor development. When hMECs were cultured on Matrigel, LMW-E mediated aberrant acinar morphogenesis, including enlargement of acinar structures and formation of multi-acinar complexes, as denoted by reduced BIM and elevated Ki67 expression. Similarly, inducible expression of LMW-E in transgenic mice generated hyper-proliferative terminal end buds resulting in enhanced mammary tumor development. Reverse-phase protein array assay of 276 breast tumor patient samples and cells cultured on monolayer and in three-dimensional Matrigel demonstrated that, in terms of protein expression profile, hMECs cultured in Matrigel more closely resembled patient tissues than did cells cultured on monolayer. Additionally, the b-Raf-ERK1/2-mTOR pathway was activated in LMW-E–expressing patient samples, and activation of this pathway was associated with poor disease-specific survival. Combination treatment using roscovitine (CDK inhibitor) plus either rapamycin (mTOR inhibitor) or sorafenib (a pan kinase inhibitor targeting b-Raf) effectively prevented aberrant acinar formation in LMW-E–expressing cells by inducing G1/S cell cycle arrest. LMW-E requires CDK2–associated kinase activity to induce mammary tumor formation by disrupting acinar development. The b-Raf-ERK1/2-mTOR signaling pathway is aberrantly activated in breast cancer and can be suppressed by combination treatment with roscovitine plus either rapamycin or sorafenib.

## Author Summary

Effective cancer treatment should include targeting not only drivers of tumorigenicity but also the downstream signaling pathways that these drivers activate. Special attention has to be given to the model systems that identify these targets and interrogating if these targets are poor prognostic indicators in patients. Using cell lines cultured on plastic and extracellular matrix (Matrigel) and comparing their proteomic profiles to breast cancer tumor samples, we demonstrated that overexpression of LMW-E is concomitant with activation of the b-Raf-ERK1/2-mTOR pathway. Using mouse models, we show that induction of LMW-E is sufficient to induce mammary tumor development *in vivo*. Next, cells established from the tumors were treated with combination therapy targeting the LMW-E/CDK2 complex and the b-Raf-ERK1/2-mTOR pathway. Results revealed that this combination therapy effectively inhibited the altered proliferation of these cells. Most significantly, we showed that breast cancer patients whose tumors overexpress both LMW-E and different components of the b-Raf-ERK1/2-mTOR pathway have the worst prognosis. In summary, through the use of multiple *in vitro* and *in vivo* model systems and translating the findings to clinical specimens, we have identified a novel targeted therapy in breast cancer patients whose tumors overexpress LMW-E.

## Introduction

Cyclin E has been extensively implicated in breast cancer [1]–[7]. The function of cyclin E is modulated via association of cyclin E with CDK2, which promotes progression of cells into S phase [8]–[10]. In addition to demonstrating genomic and transcriptional amplification of the cyclin E gene in breast cancer cells [11], our laboratory initially reported that cyclin E is cleaved by elastase into low molecular weight (LMW) isoforms in breast cancers [12], [13]. Cleavage of cyclin E occurs at two N-terminal sites of full-length cyclin E (EL), giving rise to trunk 1 [LMW-E(T1)] and trunk 2 [LMW-E(T2)] isoforms. Compared to EL, the LMW-E isoforms have higher CDK2-associated kinase activity, are more resistant to inhibition by CDK inhibitors p21 and p27, and induce higher proliferation rates when introduced into cells [14], [15]. Furthermore, examination of breast cancer patient samples revealed that approximately 27% of patients express high LMW-E protein levels as assessed by Western blot analysis, and high LMW-E expression significantly correlates with poor survival [16].

Although the connection between LMW-E and breast cancer outcome is clear, understanding of how LMW-E influences mammary tumor formation is lacking. In the mammary gland, the acinus is composed of a bilayer of luminal epithelial cells and basal myoepithelial cells; the lumen of each acinus is hollow and contains milk secretions during lactation [17], [18]. Human mammary epithelial cells (hMECs) cultured on a reconstituted basement membrane undergo cellular proliferation and differentiation to form highly organized and polarized acinar structures [19], [20]. Although this system serves as an excellent model for studying breast cancer development *in vitro*, a direct comparison of the proteomic profiles of hMECs in culture and the proteomic profiles of patient tissues has not been reported.

Most studies aimed at elucidating the action of specific proteins in breast tumorigenesis or identifying inhibitors of proteins that warrant testing in clinical trials have been conducted using the traditional two-dimensional (2D) culture. However, 2D culture do not reflect the important contribution of the tissue microenvironment both in mediation of normal breast tissue viability and in generation of the apoptotic-resistant phenotype of breast tumors. Culturing of cells in three-dimensional (3D) matrices offers several advantages over 2D culture. Culturing cells in 3D matrices allows cells to organize into structures that mimic their *in vivo* architecture, and 3D culture is particularly useful for investigating gene functions and signaling pathways in a physiologically relevant context. In 3D culture, normal and nonmalignant hMECs can be distinguished from premalignant cells: whereas normal cells become quiescent by day 10 and organize into replicas of human breast acini with correct tissue polarity and proportions [19], [20], malignant cells continue to grow, pile up, and form large, disorganized, tumor-like colonies [21]. Additionally, 3D culture is superior to 2D culture for identifying the driving oncogenic pathways in tumor cells and the critical inhibitors that warrant testing in therapeutic trials [22]–[24]. Here, we used 3D culture to elucidate the mechanisms by which LMW-E leads to progression of breast cancer, as manifested by deregulated mammary acinar morphogenesis, increased tumorigenic potential, and altered activation of targetable signal transduction pathways identified from patient samples.

Specifically, we provide evidence suggesting that the LMW-E/CDK2 complex induces breast tumor initiation and progression by disrupting the architecture of the mammary gland. Through proteomic analysis of both LMW-E-overexpressing hMECs and tumor tissue from breast cancer patients, we identify the b-Raf-ERK1/2-mTOR pathway to be critical in the tumorigenic properties of LMW-E. Consequently, we show that the disruption of the mammary gland architecture mediated by LMW-E/CDK2 can be effectively prevented by combination treatment with roscovitine (inhibitor of CDKs) plus either rapamycin (inhibitor of mTOR) or sorafenib (a pan kinase inhibitor that has activity against b-Raf). Early steps in breast tumorigenesis are characterized by enhanced proliferation of epithelial cells and deregulated acinar formation, including enlargement of acinar structures and filling of the luminal space [21]. In this study, we report that the phenotypes mediated by LMW-E during acinar development closely resemble those of human mammary epithelial cells in the early steps of breast cancer development. Additionally, inducible LMW-E expression in transgenic mice generates hyper-proliferative terminal end buds (TEBs) resulting in enhanced mammary tumor development and metastasis. Finally, through proteomic analysis, we provide evidence that breast cancer patient samples and cells cultured in 3D matrices display a high degree of concordance, thus further supporting the usefulness of this *in vitro* culture system.

## Results

### LMW-E renders hMECs tumorigenic, and LMW-E expression is selected with increased *in vivo* passaging

The presence of LMW-E in breast cancer patient samples as well as cell lines but not in normal tissues suggests that the LMW-E isoforms contribute to the development of breast cancer [13], [16], [25]. Therefore, we examined whether ectopic expression of LMW-E in a nontumorigenic cell line could render it tumorigenic. 76NE6 cells (hMECs) stably expressing vector, EL, or LMW-E (“76NE6-vector”, “76NE6-EL”, and “76NE6-LMW-E”, respectively) were injected subcutaneously into nude mice, and xenograft development was monitored. Only 7% (1/15) of the mice injected with 76NE6-EL cells developed tumors as compared with 74% (23/31) of the mice injected with the 76NE6-LMW-E cells (p<0.0001) (Table 1).

**Table 1:** LMW-E is tumorigenic.

	Tumors/Injections (%)
76NE6-vector	0/12 (0%)
76NE6-EL	1/15 (7%)
76NE6-LMW-E	23/31 (74%)
T1G2.2	5/5 (100%)
T1G2.7	5/5 (100%)
T1G3.7	5/5 (100%)
T1G3.8	5/5 (100%)
MDA-MB-468	11/11 (100%)

Athymic mice were injected subcutaneously with 1×10^7^ 76NE6 cells stably transfected with empty vector, EL, LMW-E and MDA-MB-468 cells. After 10 weeks, the tumors were removed for expansion in culture for further *in vivo* passaging and also for IHC analysis. Tumor incidence rate was estimated with exact 95% confidence intervals and Fisher's exact tests were used to compare tumor incidence rate between/among groups (*p<0.0001: 76NE6-vector vs. 76NE6-LMW-E; 76NE6-EL vs. 76NE6-LMW-E; 76NE6-vector vs. TDCs; 76NE6-EL vs. TDCs).

**Figure 1:**
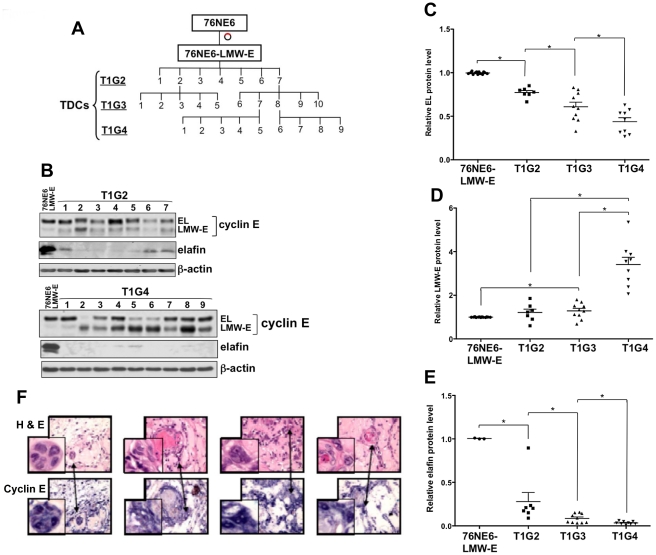
LMW-E renders hMECs tumorigenic, and LMW-E expression is selected with increased *in vivo* passaging. (A) Schematic of the generation of *in vivo* passaged clones with 3 successive injections (T1G2, T1G3, and T1G4). (B) Tumors from *in vivo* passaging were removed from mice, minced, and cultured on monolayer plates. Lysates were extracted and subjected to Western blot analysis with antibodies against cyclin E, elafin, and β-actin. EL (C), LMW-E (D), and elafin (E) protein levels were quantified by densitometry and compared between different generations of *in vivo* passaged cells (Student *t* test, *p<0.05). (F) Paraffin-embedded slides of 4 representative tumors were stained with hematoxylin and eosin (top panel) and cyclin E antibody (bottom panel).

To investigate if LMW-E expression in hMECs is sufficient to maintain tumor growth and to determine whether cells from tumors generated by LMW-E-expressing hMECs can form new tumors, LMW-E-expressing tumor cells (TDCs: tumor-derived cells) were subjected to serial *in vivo* passaging in mice. More specifically, the 76NE6-LMW-E tumors were removed for *in vitro* expansion, and two T1G2 clones were injected into mice to generate the T1G3 clones (Figure 1A). This process was repeated to generate three total generations of *in vivo* passaged clones (T1G2, T1G3, and T1G4). Interestingly, re-injection of the isolated cells from the tumors resulted in 100% tumor formation, suggesting that these cells became more tumorigenic during the process of *in vivo* passaging (Table 1).

Western blot analysis indicated that the majority of the TDCs had higher LMW-E expression than the 76NE6-LMW-E cells (Figure 1B). Furthermore, quantification of the cyclin E protein levels by densitometry indicated that *in vivo* passaging resulted in sequential reduction in the level of EL (Figure 1C) and an increase in the level of LMW-E protein with each generation of passaging (Figure 1D). The protein level of elafin (an endogenous inhibitor of the serine protease responsible for cleaving cyclin E into LMW-E isoforms [26]) also diminished with increasing passaging *in vivo*, suggesting that cyclin E was subjected to elevated proteolytic processing in the mouse microenvironment (Figure 1E). Additionally, immunohistochemical analysis of the xenograft tumors from the mice revealed strong cyclin E expression throughout the tumors and a number of the cells with enlarged nuclei and multinucleated morphology (Figure 1F). These findings suggested not only that LMW-E is tumorigenic, but also that continued expression of LMW-E provides the cells a growth advantage to promote their sustained survival in mice.

### CDK2–associated kinase activity is required for LMW-E–mediated tumorigenesis and aberrant acinar morphogenesis

**Figure 2:**
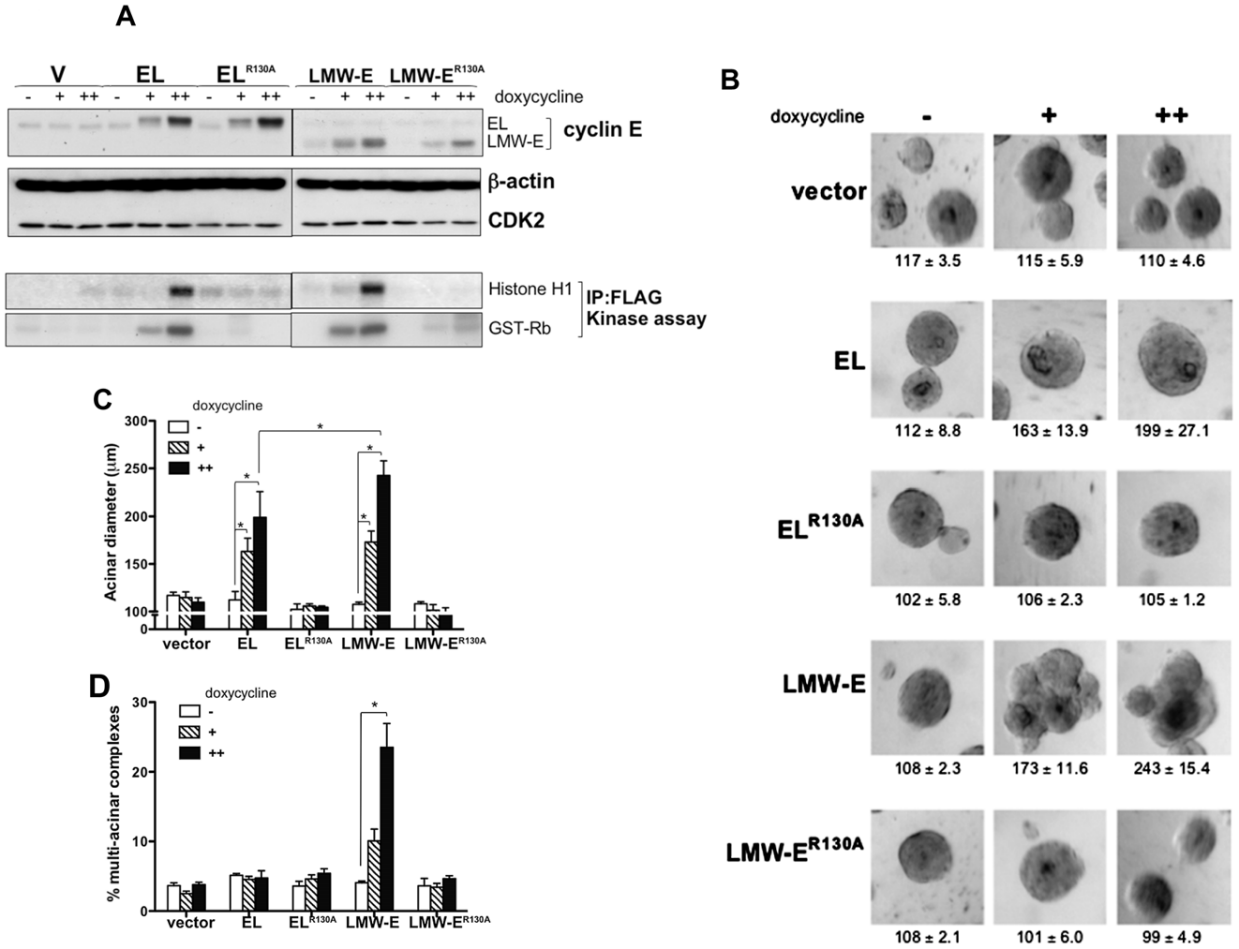
CDK2-associated kinase activity is required for LMW-E-mediated tumorigenesis and deregulation of acinar morphogenesis. (A) 76NE6-TetR cells were cultured with or without doxycycline induction, and the lysates were extracted and subjected to Western blot analysis with antibodies against cyclin E, CDK2, and β-actin. *In vitro* kinase assay was performed by immunoprecipitation with FLAG antibody and histone H1 and GST-Rb were added as substrates. Doxycycline was administered to achieve approximately 1× and 2× cyclin E protein levels. (The doxycycline concentrations for the 76NE6-TetR-V and wild-type EL and LMW-E cells were 0, 0.2, and 0.4 ng/ml, and the doxycycline concentrations for the EL^R130A^ and LMW-E^R130A^ cells were 0, 1, and 2 ng/ml.) (B) 76NE6-TetR cells were cultured on Matrigel for 15 days with or without doxycycline induction. Bright-field images were taken at day 15. Values underneath each figure represent mean diameter (µm) ± SEM. (C) The diameters of at least 100 acini from 3 different experiments were measured. Error bars = SEM (Wilcoxon rank-sum test, *p<0.05). (D) Multi-acinar complexes were counted. Error bars = SEM (Wilcoxon rank-sum test, *p<0.05). Multi-acinar complexes were defined as complexes with more than 2 acini growing on top of each other. Logistic regression models were used to compare the rate of formation of multi-acinar complexes between/among groups (*p<0.05).

To examine the role of CDK2 in LMW-E-mediated tumorigenesis, we generated another model system, as previously described [27] in which the expression of FLAG-tagged vector, EL, and LMW-E in 76NE6 cells could be induced by varying doxycycline concentrations (Figure 2A). *In vitro* kinase assay using histone H1 and GST-Rb as substrates confirmed that inducible EL, and LMW-E, had functional cyclin E-associated kinase activity (Figure 2A). We injected the 76NE6 cells with inducible protein expression subcutaneously into nude mice and induced the expression of vector, EL, and LMW-E with doxycycline 24 hours later. The tumor incidence rates were significantly higher in mice treated with 500 µg/ml doxycycline than in mice not treated with doxycycline by Fisher exact test (p<0.0001) (Table 2). In addition, LMW-E induction with 500 µg/ml doxycycline led to tumor formation in more than 90% of the injections, whereas EL induction with 500 µg/ml doxycycline led to tumor formation in only 17% of mice (Table 2). The tumor incidence rate mediated by LMW-E in this xenograft model is consistent with the transgenic model of LMW-E overexpression previously reported [28].

**Table 2:** The tumorigenicity of LMW-E requires CDK2–associated kinase activity.

Dox (µg/ml)	0	500
	Tumors/Injections (%)	Tumors/Injections (%)
vector	1/18 (6)	2/18 (11)
EL	2/18 (11)	3/18 (17)
LMW-E	2/18 (11)	19/20 (95)
EL^R130A^	0/8 (0)	1/12 (8)
LMW-E^R130A^	0/8 (0)	2/12 (17)

Athymic mice were injected with 1×10^7^ 76NE6-TetR cells with inducible expression for empty vector, EL, LMW-E, EL^R130A^, and LMW-E^R130A^. Doxycycline was added to drinking water containing 1% sucrose 24 hours after injection and replaced twice weekly. The diameter of the tumors were measured and recorded weekly. Tumor incidence rate was estimated with exact 95% confidence intervals and Fisher's exact tests were used to compare tumor incidence rate between/among groups (*p<0.0001: LMW-E 0 vs. LMW-E 500; vector 500 vs. LMW-E 500; EL 500 vs. LMW-E 500; LMW-E 500 vs. EL^R130A^ 500; LMW-E 500 vs. LMW-E^R130A^).

Since cyclin E is the regulatory subunit of the cyclin E/CDK2 complex and is enzymatically inactive when unbound, we speculated that the oncogenicity of LMW-E requires interaction with CDK2. Consequently, we generated a point mutation at R130A in the cyclin E gene that prevents cyclin E from interacting with CDK2, thereby suppressing the cyclin E/CDK2 kinase activity [29]. The CDK2-associated kinase activity of these inducible mutants was compromised as indicated by lack of histone H1 and GST-Rb phosphorylation (Figure 2A). To determine whether cyclin E-mediated tumorigenesis is dependent on the kinase activity associated with CDK2, we injected 76NE6 cells with inducible expression of EL^R130A^ and LMW-E^R130A^ into nude mice. The resulting tumor incidence was 17% or less for cells expressing EL^R130A^ and LMW-E^R130A^ (Table 2) indicating that CDK2-associated kinase activity is necessary for LMW-E-mediated tumorigenicity. These results demonstrated that cells expressing LMW-E have a higher frequency of tumor formation than cells expressing EL, and this oncogenicity is critically dependent on the CDK2-associated kinase activity. This observation is consistent with our recently published results in which we reported that LMW-E overexpression does not induce mammary tumor development in CDK2^−/−^ transgenic mice [28].

We next asked if deregulation of acinar development is responsible for LMW-E-mediated oncogenicity. Examination of acinar development of hMECs cultured on a reconstituted basement membrane revealed that while induced EL expression led to generation of large acini with the typical spherical structure, induced LMW-E expression led to generation of large acini with irregular shapes (Figure 2B). Quantification of the size of the acini revealed that deregulation of acinar morphogenesis by LMW-E was dose dependent, with higher cyclin E expression generating larger acini (Figure 2C). In contrast, induction of EL^R130A^ and LMW-E^R130A^ did not increase acinar size (Figure 2B and 2C). Furthermore, only wild-type LMW-E expression generated multi-acinar complexes (complexes with multiple acini forming aggregate structures), a phenotype not observed with EL, EL^R130A^ and LMW-E^R130A^ expression (Figure 2D). Overall, our data suggested that LMW-E depends on CDK2-associated kinase activity to induce mammary tumorigenesis and aberrant acinar morphogenesis.

### LMW-E induces formation of large and highly proliferative acini

**Figure 3:**
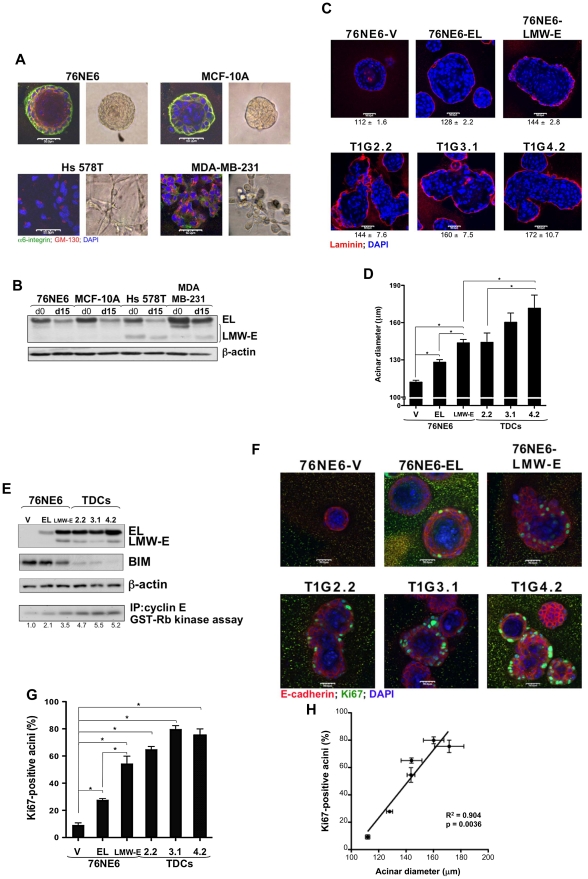
LMW-E induces formation of large and highly proliferative acini. (A) 76NE6, MCF-10A, HS 578T, and MDA-MB-231 cells were seeded at a density of 70 cells/mm^2^ on 1-mm-thick Matrigel. After 15 days in 3D culture, cells were fixed and immunostained with GM-130 and α6-integrin antibodies. Nuclei were counterstained with DAPI. Scale bar = 50 µm. (B) Lysates from these cells were isolated at days 0 (d0) and 15 (d15) of acinar morphogenesis and subjected to Western blot analysis with the indicated antibodies. (C and D) 76NE6 cells stably transfected with vector, EL, and LMW-E and tumor-derived cells (TDCs) were subjected to analysis similar to that in (A) (V = vector). Scale bar = 50 µm. The diameters of the acini were measured and averaged from 3 independent experiments. Values underneath each figure represents mean (µm) ± SEM. Error bars = SEM (Student *t* test, *p<0.05). (E) Lysates from these cells were isolated at day 15 and subjected to Western blot analysis with the indicated antibodies. *In vitro* kinase assay was performed by immunoprecipitation of lysates from 3D culture using polyclonal cyclin E antibody and incubation with (γP32)ATP and GST-Rb. (F and G) Cells cultured on Matrigel for 15 days were fixed and immunostained with E-cadherin and Ki67 antibodies. Nuclei were counterstained with DAPI. Scale bar = 50 µm. The number of Ki67-positive cells per acinus was counted and averaged from 3 independent experiments. Error bars = SEM (Student *t* test, *p<0.05). (H) Linear regression of the correlation between acinar diameter and percentage of Ki67-positive cells.

The 3D cell culture system can be used to distinguish nonmalignant from malignant cells on the basis of the phenotypes observed [19]. 76NE6 cells and MCF-10A cells (immortalized hMECs) formed polarized acinar structures when cultured on Matrigel as indicated by α6-integrin staining on the basal surface and GM-130 staining on the apical surface (Figure 3A). In contrast, breast cancer cell lines such as Hs 578T and MDA-MB-231, which express endogenous LMW-E (Figure 3B) did not form coherent acini and demonstrated disordered polarity as indicated by unorganized α6-integrin and GM-130 staining (Figure 3A).

Using 76NE6 cells with stable vector, EL, and LMW-E expression, we found that, similar to what we observed in cells with inducible protein expression (Figure 2B), overexpression of EL led to generation of large but still spherical acini, while overexpression of LMW-E led to generation of large, irregularly shaped structures and multi-acinar complexes (Figure 3C). Aberrant acinar development was also observed in the TDCs, in which the acini were approximately 28% larger than the structures formed by the 76NE6 cells with vector expression (p<0.05) (Figure 3D).

During normal acinar morphogenesis, cells are highly proliferative and then undergo apoptosis of the lumen with subsequent proliferative arrest and induction of differentiation by day 15 in culture [30]. As expected, the 76NE6 cells arrested proliferation by downregulating cyclin E in 3D culture (Figure 3B). However, cyclin E protein levels in the 76NE6-LMW-E cells and in the TDCs were upregulated during acinar morphogenesis compared to the cyclin E protein levels in the 76NE6-V and 76NE6-EL cells (Figure 3E). Moreover, the cyclin E-associated kinase activity of the LMW-E-expressing cells was also elevated, suggesting that cells in these acinar structures were still actively proliferating, passing through the G1/S-phase checkpoint and thus leading to formation of enlarged acini. We also observed that the levels of cyclin E protein as well as mRNA transcript were much higher in the 76NE6-LMW-E cells compared to the 76NE6-EL cells (Figure 3E and Figure S1A), which is a phenomenon that was also observed in the transgenic mouse model with overexpression of LMW-E (Figure S1B). To test if overexpression of LMW-E in the transgenic mice upregulates the endogenous mouse cyclin E gene, we analyzed mouse cyclin E mRNA expression levels in the tumor and the contralateral mammary gland of 3 different LMW-E-overexpressing transgenic mice (Figure S1B). Quantitative RT-PCR analysis showed a 3-fold increase in the abundance of endogenous cyclin E mRNA in the tumors when compared to the contralateral mammary glands. These results are consistent with a model in which, during tumor progression, LMW-E expression activates a positive feedback loop leading to increase expression of endogenous cyclin E.

BIM, a member of the Bcl-2 pro-apoptotic family, has been shown to be responsible for cell death during late acinar morphogenesis to generate a hollow lumen in the acinus [31]. We found that BIM protein levels were downregulated in the LMW-E-expressing acini, suggesting that these cells bypass morphogenetic cues that cause growth arrest and apoptosis of the luminal cells (Figure 3E). To determine whether LMW-E expression was sufficient to prevent growth arrest of cells in mature acini, we fixed acini at 15 days and stained them for Ki67. While Ki67 expression was not detectable in the 76NE6-V acini, LMW-E-expressing acini displayed high Ki67 staining, particularly in cells that were in contact with the basement membrane (Figure 3F and 3G). Furthermore, we determined a strong positive correlation between the acinar diameter and the percentage of Ki67-positive acini, indicating that the formation of large acini may be due to increased proliferation (Figure 3H). Collectively, these findings provided evidence that expression of LMW-E is sufficient to induce generation of large and misshapen acini that exhibit enhanced cell proliferation and decreased apoptosis. These phenotypes resemble those observed in ductal carcinoma *in situ* and also those caused by ErbB2 activation [21] and may explain the high tumorigenic potential of LMW-E over EL.

### LMW-E induces ductal hyperplasia *in vivo* and invasion in Boyden chamber assays

**Figure 4:**
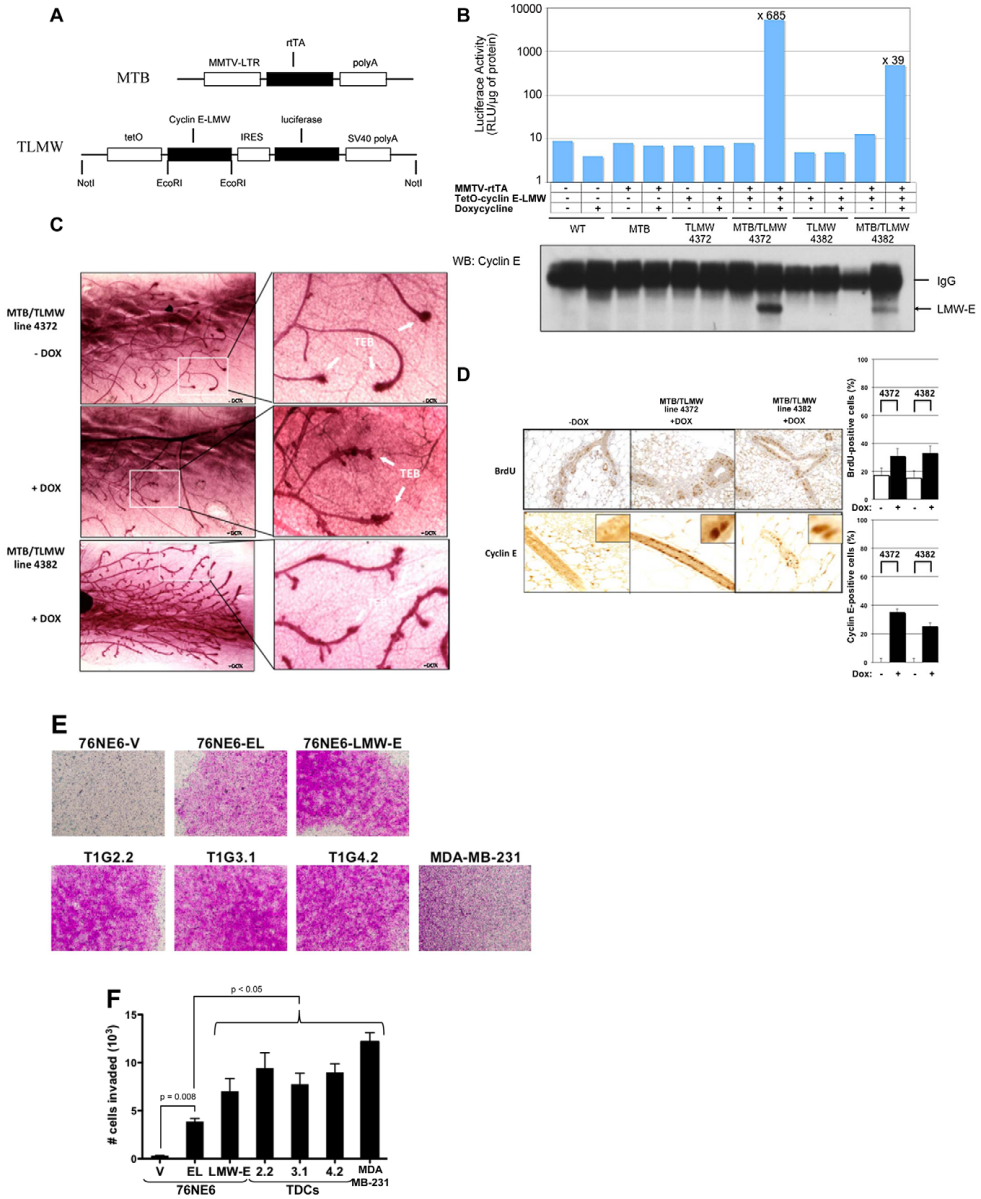
LMW-E induces ductal hyperplasia in vivo and invasion in Boyden chamber assays. (A) Doxycycline-dependent expression of LMW-E in MTB/TLMW mice induces ductal hyperplasias. Bi-transgenic mice carrying both the MMTV-rtTA-pA (MTB) transgene and the TetO-LMW-E (TLMW) transgene express the rtTA transactivator in the mammary epithelium but do not express LMW-E unless doxycycline is added. (B) Virgin female mice (6 weeks old) of the indicated genotypes were either left untreated or administered 2 mg/ml doxycycline in drinking water for 4 days. Mammary gland extracts were prepared and subjected to luciferase activity assay and Western blot analysis of LMW-E expression. (C) Whole-mount staining of mammary glands from 8-week-old mice either left untreated (upper panels) or administered 2 mg/ml doxycycline in drinking water for 7 days (lower panels). TEB, terminal end buds. (D) Representative BrdU and cyclin E analyses performed on mammary sections from MTB/TLMW 6-week-old females administered doxycycline for 4 days (left panels, magnification: ×200). BrdU incorporation and percentage of cyclin E-positive cells were quantified (right panels) by counting 1,000 cells per section in 3 mice. (E) Boyden Chamber assays. 76NE6 parental and different EL, LMW-E and TDC variants were seeded on Matrigel-coated transwell chamber and incubated on top of fibronectin-containing media for 24 hours. The cells on top of the membrane were removed and the cells remaining on the bottom were stained with crystal violet and images were taken with a light microscope. (F) After 24 hours incubation, the cells on the bottom of the transwell were collected and counted. Statistical analysis used was unpaired student's *t*-test. MDA-MB-231 cells were used as positive control for the invasion assay.

Having shown that LMW-E expression renders hMECs tumorigenic and leads to altered acinar morphogenesis, we set out to determine whether there was a direct cause-and-effect relationship between induction of LMW-E expression and altered mammary ductal structures in a transgenic mouse model. We developed transgenic mice with doxycycline-inducible LMW-E expression and examined these mice for altered TEB formation in the mammary gland and tumorigenesis in response to induction of LMW-E expression (Figure 4A). Following 4 days of doxycycline treatment, MTB/TLMW mice demonstrated a 685-fold increase in luciferase activity above background for line 4372 and about 39-fold above background for line 4382 and LMW-E protein expression was detected by Western blot analysis; however, in MTB/TLMW mice not treated with doxycycline and in doxycycline-treated MTB or TLMW mice, no increase in luciferase activity or LMW-E protein expression was observed (Figure 4B). Morphological examination of carmine-stained whole mounts revealed striking hyperplastic abnormalities in mammary ductal trees of both MTB/TLMW lines of mice with induced expression of LMW-E (Figure 4C, lower panels). The mammary glands of these mice displayed abnormal development, including the formation of solid cellular masses along the primary ducts that resembled abortive side buds and misshapen TEBs. In contrast, mammary tissues from MTB/TLMW mice without induced expression of LMW-E were histologically indistinguishable from tissues from wild type and MTB mice and had normal club-shaped TEBs (Figure 4C, upper panels). In addition, the mammary epithelium of both MTB/TLMW lines with induced LMW-E expression showed 2-folds higher in BrdU incorporation as compared to the mammary epithelium of MTB/TLMW mice without induced LMW-E expression (p = 0.03) indicating that LMW-E overexpression, as shown by immunohistochemistry (Figure 4D; 34.9%±2.7% cyclin E-positive cells for line 4372 and 25.1%±1.6% cyclin E-positive cells for line 4382), induces high proliferation in the mammary epithelium. These data obtained from the transgenic mice suggested that inducible LMW-E expression in the mouse mammary epithelium results in hyper-proliferation and aberrant acinar morphogenesis similar to what was observed with the hMECs expressing LMW-E cultured on Matrigel in the xenograft model system.

We have shown previously that approximately 25% of transgenic mice with LMW-E expression developed metastasis as compared to 8.3% of tumors with EL overexpression [32]. Cellular invasion is one of the critical events leading to successful metastasis and requires migration of the tumor cells through the basement membrane to invade the surrounding tissues [33]. The Boyden chamber invasion assay was performed to investigate whether LMW-E expression in hMECs enhances cellular invasiveness. The cells were seeded on a microporous transwell insert on top of a thin layer of Matrigel with fibronectin on the other side of the membrane to act as a chemo-attractant. After 24 hours, the cells that have invaded to the bottom side of the membrane were stained with crystal violet for visualization. Figure 4E shows that while the vector control cells were unable to invade through the Matrigel basement membrane, cells with cyclin E expression were highly invasive. More specifically, quantification of the invaded cells demonstrate that while all cells with cyclin E expression invaded through the basement membrane significantly more than vector control cells, cells with LMW-E expression invaded significantly more than cells with EL expression (p<0.05) (Figure 4F). Collectively, we provide evidence suggesting that overexpression of LMW-E enhances the invasiveness of hMECs.

### High LMW-E expression is associated with the activated b-Raf-ERK1/2-mTOR pathway *in vitro* and in human tumor tissues

**Figure 5:**
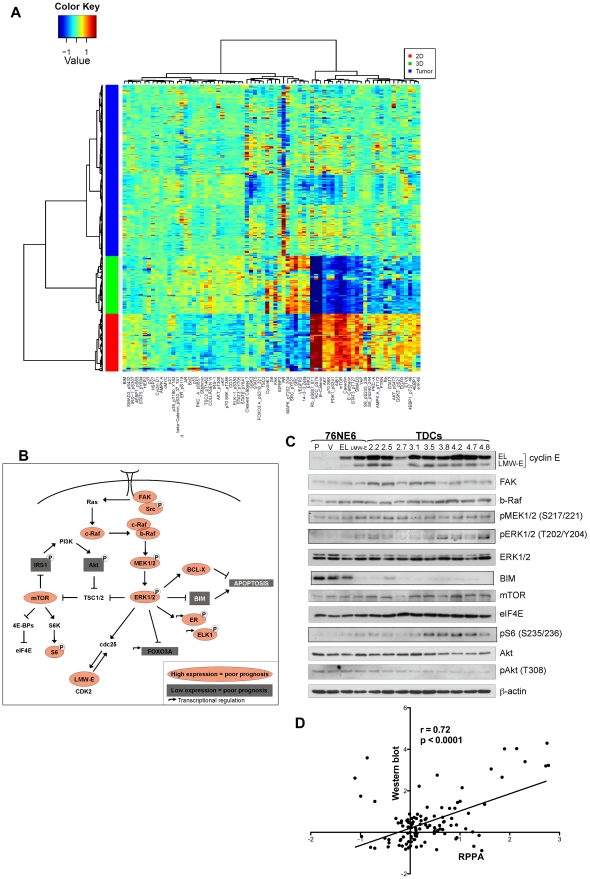
High LMW-E expression is associated with activated b-Raf-ERK1/2-mTOR pathway *in vitro* and in patient tissues. (A) Hierarchal cluster analysis of protein expression in 76NE6, 76NE6-LMW-E and all of the LMW-E-expressing tumor clones grown on 2D (red) and 3D (green) cultures and 276 breast cancer patient samples (blue). (B) Proteins whose expression was associated with high LMW-E levels. Red indicates that high LMW-E along with high protein expression was associated with poor prognosis; grey indicates that high LMW-E along with low protein expression was associated with poor prognosis. (C) The lysates from 3D culture were subjected to Western blot analysis to validate the RPPA data. The cell lines are 76NE6-parental (P) and with stable expression of vector (V), EL, and LMW-E and the tumor-derived cells (TDCs). (D) Linear regression analysis of the RPPA data and the densitometry values of all the proteins analyzed by Western blot analysis in Figure 5C. The same antibodies were used for the two types of analysis.

While it is widely accepted that the 3D culture system serves as a more physiologically relevant model for the investigation of cell behavior compared to 2D plastic surface [18], [20], no direct comparison between cells cultured on this 3D model and human samples has been performed. Therefore, we next aim to compare the protein expression patterns between cells grown on 2D culture, 3D culture and human breast tumor tissues. The reverse-phase protein array (RPPA) assay was used to compare the expression levels of 73 different proteins involved in major signaling transduction pathways and cellular processes between xenografted tumor-derived cells (TDCs) grown on 2D monolayer and in 3D Matrigel cultures and 276 human breast cancer samples previously described [16]. The RPPA method is a proteomic protein expression analysis that has been shown to be highly reproducible in analyzing the expression patterns of proteins involved in cell signaling [34]–[36]. For these analyses, serially diluted lysates prepared from cell lines cultured on 2D and 3D as well as from 276 tumor specimen were arrayed on nitrocellulose-coated slides as described previously [16]. Each slide was then probed with a validated primary antibody plus a biotin-conjugated secondary antibody. Table S1 lists the antibody targets used for this study, which were selected as being relevant to breast cancer through a literature review. Hierarchical clustering was then performed using Euclidean distance and Ward's minimum variance for agglomeration (Figure 5A). The resulting heat map demonstrated that the cells from 2D and 3D cultures had strikingly different protein expression patterns and that the protein expression pattern of the cells from 3D cultures more closely resembled that of patient tissues than did the protein expression pattern of cells grown on monolayer (Figure 5A). Most of the proteins that show a distinct expression pattern between 2D and 3D cultures play key roles in cell proliferation, specifically, the G1 to S transition (Table S2). These results were expected since it has been established that the 3D culture system is a more physiologically relevant model than cell culture on a 2D plastic surface for the investigation of cellular behavior [18], [20]. Furthermore, in an unsupervised analysis of the patient RPPA data, we observed separate clustering between the low and high LMW-E-expressing breast tumors but not between low and high full-length cyclin E (Figure S2A and S2B).

We next identified the proteins whose expression was significantly associated with LMW-E levels as well as patient survival in the tumor database (Figure 5B). Our analysis revealed that the b-Raf-ERK1/2-mTOR pathway is activated in the breast cancer patient samples as well as in the tumor cells cultured on Matrigel with high LMW-E expression (Figure 5B and 5C and Table S3). Furthermore, a direct comparison between the levels of all the proteins analyzed in Figure 5C by Western blot and those obtained from the RPPA analysis showed high concordance (Pearson coefficient = 0.723, p<0.001) and also validated the activation of this signaling axis *in vitro* (Figure 5C and 5D). Additionally, breast cancer patient tumors with high LMW-E expression also expressed high levels of b-Raf, pMEK1/2 (S217), ERK2, mTOR, and eIF4E and a low level of pAkt (T308) (Table 3 and Figure S3). Collectively, these data suggested that in terms of proteomic expression patterns, breast cancer cells grown in 3D culture more closely resemble human tumors than do breast cancer cells grown in 2D culture thereby underscoring the usefulness of this *in vitro* model system.

**Table 3:** Patient protein expression based on low and high LMW-E and EL levels by RPPA analysis.

	Relative protein levels		
Proteins	Low LMW-E	High LMW-E	P value*
ERK2			2.27E-07
Median	0.47	0.542	
Mean (range)	0.459 (0.134–1.02)	0.581 (0.134–1.88)	
pMEK1/2 (S217)			4.85E-06
Median	0.2	0.223	
Mean (range)	0.203 (0.111–0.348)	0.235 (0.112–0.806)	
b-Raf			2.05E-04
Median	0.159	0.209	
Mean (range)	0.198 (0.0549–0.756)	0.264 (0.069–1.505)	
mTOR			5.45E-04
Median	0.099	0.127	
Mean (range)	0.112 (0.043–0.297)	0.135 (0.0424–0.423)	
eIF4E			7.06E-02
Median	0.356	0.392	
Mean (range)	0.381 (0.143–0.777)	0.405 (0.093–0.795)	
pAkt (T308)			4.67E-02
Median	0.145	0.123	
Mean (range)	0.164 (0.071–0.841)	0.168 (0.068–1.362)	

	Low EL	High EL	
ERK2			0.2
Median	0.516	0.53	
Mean (range)	0.521 (0.134–1.344)	0.568 (0.284–1.88)	
pMEK1/2 (S217)			0.5
Median	0.212	0.216	
Mean (range)	0.222 (0.111–0.806)	0.22 (0.143–0.352)	
b-Raf			2.09E-05
Median	0.163	0.246	
Mean (range)	0.223 (0.055–1.505)	0.292 (0.089–0.752)	
mTOR			1.29E-02
Median	0.111	0.13	
Mean (range)	0.121 (0.042–0.423)	0.142 (0.047–0.297)	
eIF4E			5.65E-03
Median	0.362	0.416	
Mean (range)	0.384 (0.093–0.795)	0.44 (0.143–0.703)	
pAkt (T308)			0.9
Median	0.131	0.134	
Mean (range)	0.162 (0.071–1.227)	0.184 (0.068–1.362)	

*: Wilcoxon ranksum test.

### Combination drug treatment prevents induction of aberrant acinar development by LMW-E

**Figure 6:**
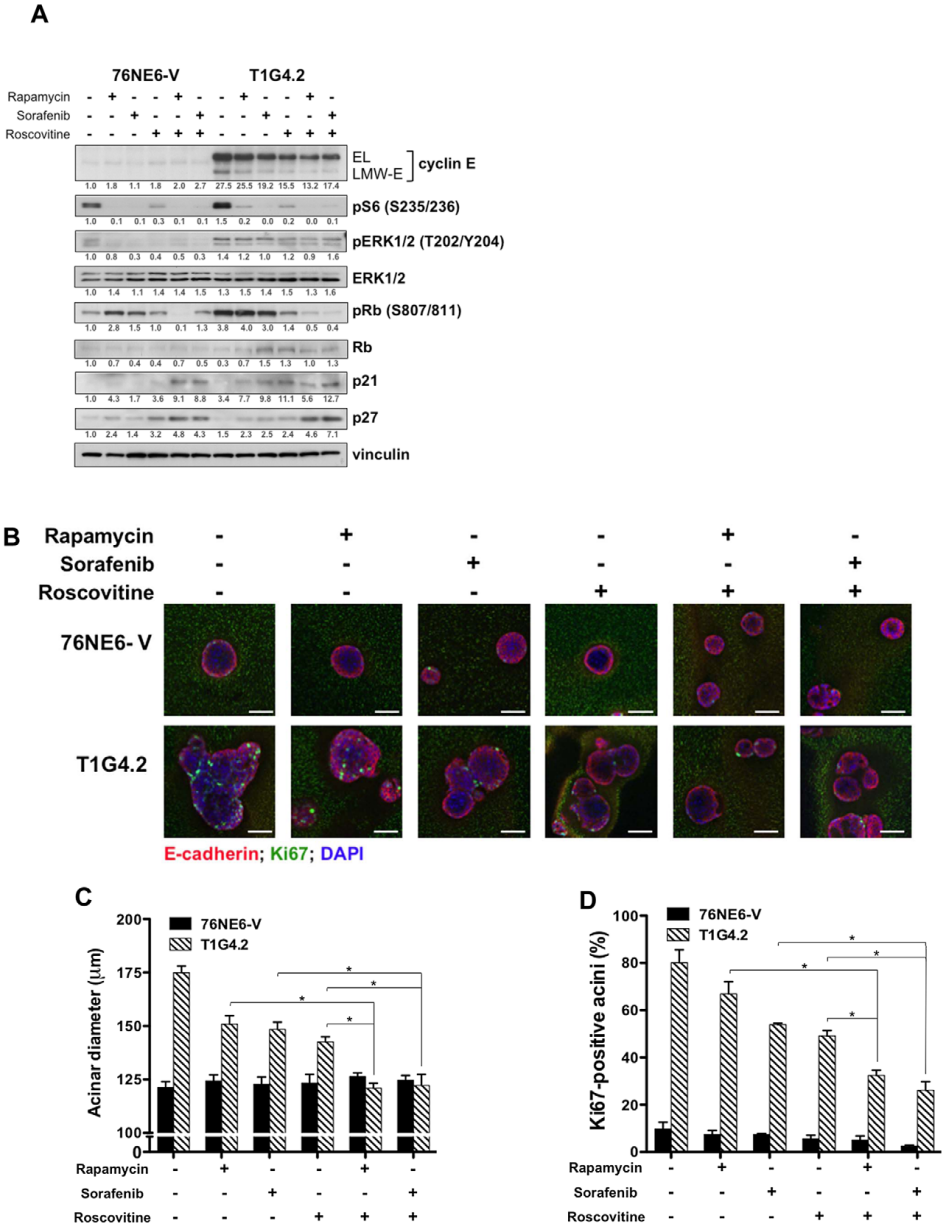
Combination drug treatment prevents induction of aberrant acinar development by LMW-E. (A) Cells were seeded on Matrigel for 24 hours and then treated with rapamycin, sorafenib, and roscovitine as indicated. Medium containing drugs was replaced every 4 days, and lysates were collected on day 15 for Western blot analysis with the indicated antibodies. (B) On day 15 of Matrigel culture, cells grown as in (A) were fixed and stained with E-cadherin (red) and Ki67 (green), and nuclei were counterstained with DAPI (blue). Scale bar = 50 µm. (C) The diameters of the acini were measured and averaged from three independent experiments. Error bars = SEM (Student *t* test, *p<0.05). (D) The number of Ki67-positive cells per acinus was counted and averaged from three independent experiments. Error bars = SEM (Student *t* test, *p<0.05). Kaplan-Meier survival plots demonstrating association between full length and high LMW-E on disease-specific survival. Association of:

Having established the importance of the CDK2-associated kinase activity in aberrant acinar morphogenesis in 3D culture and given that the b-Raf-ERK1/2-mTOR signaling axis was deregulated in tumor cells and patient samples with high LMW-E expression, we hypothesized that combination treatment with roscovitine (a CDK inhibitor) plus either rapamycin (an mTOR inhibitor) or sorafenib (a pan kinase inhibitor that has activity against b-Raf) can prevent the induced-aberrant acinar morphology. Combination treatments of cells cultured in Matrigel using these agents resulted in a larger reduction of the levels of pS6 (S235/236), pERK1/2 (T202/Y204), and pRb (S807/811) than no treatment or treatment with single agents (Figure 6A). Moreover, the combination treatments upregulated the expression of the CDK inhibitors p21 and p27, consistent with a cell cycle arrest at the G1-S phase. Examination of the acinar formation as a result of the combination drug treatments revealed that the TDCs displayed a significant reduction in acinar size and Ki67 levels compared to the untreated cells and cells treated with single agents (Figure 6B and 6D, Figure S4, Figure S5A and S5B, and Tables S4 and S5). In contrast, the 76NE6-V and 76NE6-EL cells displayed no change in these phenotypes in response to the drug treatments, suggesting that the absence of LMW-E expression may protect these cells from the toxic effects of the drugs. Thus, roscovitine in combination with either rapamycin or sorafenib can prevent the development of the aberrant acinar phenotypes caused by LMW-E expression, confirming a role for LMW-E/CDK2 kinase activity in causing formation of large, multilobular acini and demonstrating a potential therapeutic approach to treat cancer patients with high LMW-E expression.

### Activated b-Raf-ERK1/2-mTOR signaling pathway and high LMW-E expression predict poor survival

**Figure 7:**
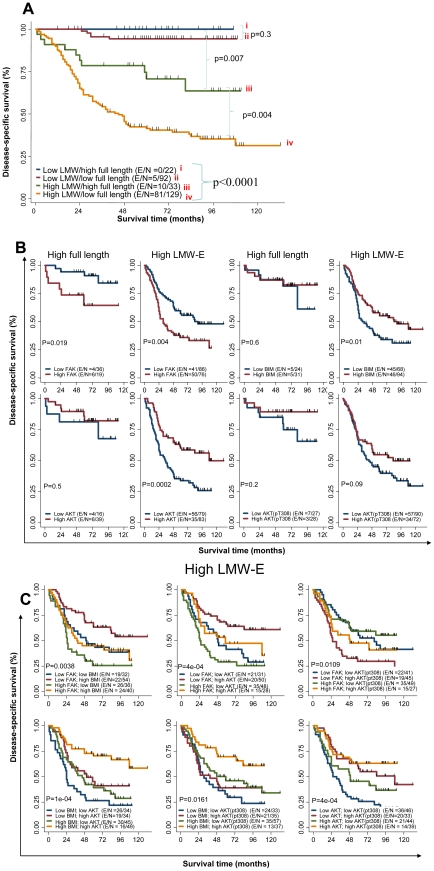
Activated b-Raf-ERK1/2-mTOR signaling pathway and high LMW-E expression predict poor survival in breast cancer patients. Kaplan-Meier survival plots demonstrating association between full length (EL) and high LMW-E on disease specific survival. Association of (A) LMW-E and full length level, (B) Combination of FAK, BIM, Akt and pAkt (T308) protein levels in patients with high full length or high LMW-E, (C) FAK, BIM, Akt and pAkt (T308) protein levels in patients with high LMW-E. The expression levels of FAK, BIM, Akt and pAkt (T308) were dichotomized using their median values from all 276 patient samples. E (events)/N (total number), refer to number of patients who die of breast cancer/total number of patients in the group being analyzed. All p values are based on log-rank test. The overall p value of comparison amongst the 4 different groups is p<0.0001.

In a large retrospective clinical study, we previously found that breast cancer patients whose tumors had high levels of LMW-E expression, as determined by Western blot analysis, have significantly worse DSS than patients whose tumors had low LMW-E expression [16]. In the study reported herein, we used tissue samples from 276 of these patients for RPPA analysis to investigate large-scale protein expression pattern. The 276 patients were divided into 4 groups based on both LMW-E and EL expression and subjected to Kaplan-Meier analysis (Figure 7A). The four groups consisted of (i) 22 patients with low LMW-E/high EL, (ii) 92 patients with low LMW-E/low EL, (iii) 33 patients with high LMW-E/high EL, and (iv) 129 patients with high LMW-E/low EL. Similar to our previous observation, we found that patients with high LMW-E protein levels had significantly worse DSS than patients with low LMW-E expression (p<0.0001) (Figure 7A). More specifically, only patients whose tumors overexpress LMW-E (groups iii and iv) regardless of whether or not they also overexpress EL, have a poor prognosis (Figure 7A). Additionally, those patients whose tumors overexpress EL, in the absence of any LMW-E (group i) have the best prognosis. This new analysis clearly indicated that LMW-E overexpression, but not EL, is responsible for poor patient outcome.

Next, we performed bivariate analysis of cyclin E level along with key nodes in the b-Raf-ERK1/2-mTOR pathway, which revealed that among breast cancer patients with high LMW-E expression, those with high FAK levels had significantly worse DSS than those with low FAK levels (p = 0.0042) (Figure 7B and Figure S6). In contrast, among patients with high LMW-E expression, low BIM or low total Akt levels were associated with worse survival. Additionally, the overall DSS of patients with high LMW-E combined with these proteins in the b-Raf-ERK1/2-mTOR pathway was dramatically worse than in the patients with high EL expression (Figure 7B and Figure S6).

To determine whether these individual proteins collaborate to reduce patient survival, we performed multivariate analysis by analyzing patients with high LMW-E expression and combining 2 additional proteins. We found that patients with high LMW-E, high FAK, and low BIM, Akt, or pAkt (T308) experienced significantly worse DSS than the opposite groups (p<0.05) (Figure 7C). In addition, patients with high LMW-E, low BIM, and low Akt or pAkt (T308) experienced significantly worse DSS (p<0.05). Interestingly, we were not able to find statistical significance between EL expression in the same multivariate analysis with these proteins (Figure S7). Essentially, our statistical analysis suggests that it is likely that LMW-E, FAK, BIM, Akt, and pAkt (T308) function in the same pathway to adversely affect patient survival with breast cancer.

## Discussion

There is mounting evidence suggesting that the LMW-E isoforms play a unique role in mammary tumorigenesis. Our current understanding of cell cycle deregulation by LMW-E consists of enhanced S-phase entry [13], aberrant centrosomal amplification [37], and genomic instability [38]. In this report, we utilized three model systems (xenograft transplantation, 3D acinar morphogenesis, and an inducible transgenic mouse model) that recapitulate the human mammary gland to examine the tumor-initiating potential of LMW-E.

We first demonstrated that LMW-E has greater oncogenic potential than EL, as indicated by tumor-initiating activity in nude mice with subcutaneous xenografts. Moreover, LMW-E expression is selected with increasing *in vivo* passaging suggesting that LMW-E provides a growth advantage in tumors. Indeed, selective pressure exerted from the *in vivo* microenvironment has previously been shown to favor further genetic and epigenetic alterations that eventually progress to highly advanced tumor stages [39]. Additionally, the inducible transgenic mouse model system provided evidence for a direct role of LMW-E in mediating alteration in the TEBs in the mammary gland, which is required for tumor generation in these mice. Furthermore, this model system underscores the important role of the microenvironment in the development of morphological characteristics and growth patterns.

We observed an interesting phenomenon in which tumor cells with LMW-E expression and transgenic mice with inducible LMW-E expression demonstrated an elevation in the level of EL expression. We speculate that high LMW-E protein levels may lead to hyperactive G1-S transition causing a positive feedback loop acquired during tumor progression that activates the transcription of the endogenous cyclin E mRNA through activation of E2F. Increased E2F activity has been shown to stabilize cyclin E by reducing conjugation with ubiquitin [40]. Additionally, cyclin E transcription has been reported to be positively regulated by the E2F transcription factor, and in fact, the cyclin E promoter does contain several E2F binding sites [41]. Indeed, this observation warrants further investigation into the transcriptional regulation of cyclin E expression and the possible positive feedback loop that is critical for mammary tumorigenesis.

The acinar morphogenesis assay has been widely used to model the early stages of mammary oncogenesis [19], [21]. Our data suggest that LMW-E may exert its tumorigenic potential via disruption of the acinar morphogenetic process resulting in larger and misshapen acini due to failure of proliferation arrest and apoptotic induction [30]. High Ki67 expression in the cells on the outer layer of the acini suggests continued proliferation that likely leads to disruption of the spherical integrity of the structures. These aberrant morphological phenotypes mediated by LMW-E are similar to the characteristics described for ductal carcinoma *in situ* and may explain the role of LMW-E in mammary oncogenesis.

The fact that LMW-E requires CDK2 kinase activity to drive multiacinar complexes and promote tumor-initiating activity of hMECs in mice suggests that LMW-E itself has no intrinsic oncogenic activity. This observation corroborates with our recent publication demonstrating that CDK2 is necessary for LMW-E-mediated mammary tumor formation in transgenic mice [28]. Therefore, treatment of tumors with high LMW-E protein levels can be achieved by inhibiting CDK2 kinase activity. Roscovitine is a promising agent for targeting multiple types of tumors, including breast cancer, sarcoma, non-small cell lung cancer, multiple myeloma, and lymphoma [42]–[46]. In fact, treatment of the mice with LMW-E-induced tumor using two different CDK inhibitors, meriolin and roscovitine, significantly delayed mammary tumor formation by approximately 6 weeks [28]. In this study, we also demonstrated that combination treatment using roscovitine together with rapamycin or sorafenib of LMW-E-expressing acini efficiently prevents the aberrant morphogenetic phenotypes without toxic effects on hMECs lacking LMW-E expression. These observations implicate an effective therapeutic strategy of inhibiting the CDK2-associated kinase activity and perhaps combining it with rapapmycin or sorafenib to treat breast cancer patients with high LMW-E expression.

The results from the proteomic analysis demonstrated a marked contrast in the protein expression profiles of cells grown on monolayer and cells grown in 3D culture and illustrated a high similarity between cells in 3D culture and human tumor tissues, thus establishing a bridge between the 3D culture system and human tissues and further supporting the use of this culture system for biological study [47]. In fact, gene expression signatures of mammary cells extracted from this 3D culture system can be reliably used to predict patient outcome in which the signature of growth-arrested and well-organized hMECs predicts favorable clinical outcome [48], [49]. Data from this study also allowed for the delineation of a signaling pathway that is deregulated in breast cancer patients who express high LMW-E levels. We demonstrated that tumors and cell lines with high LMW-E expression have upregulated b-Raf-ERK1/2-mTOR signaling, which has been reported to result in enhanced cell survival and reduced apoptosis [50]–[52]. Future pre-clinical studies will be aimed at examining if human breast tumors with high LMW-E expression are selectively sensitive to combination therapy with roscovitine, and sorafenib or rapamycin as compared with those without high LMW-E. These studies will help establish the clinical relevance of LMW-E expression as a marker for the targeted therapies identified in this report.

In summary, LMW-E/CDK2 deregulates mammary acinar development, leading to enlarged and misshapen structures. Failure of LMW-E-expressing acini to arrest proliferation and undergo luminal apoptosis suggests upregulation of signaling involving cell survival and growth by hyperactive LMW-E/CDK2 complexes. Our data suggest that the combination of roscovitine with either rapamycin or sorafenib should be evaluated as a therapeutic strategy to treat breast cancer patients with high LMW-E expression.

## Methods

### Constructs and cell culture

All immortalized cell lines were cultured in DFCI-1 medium as described previously [53]. FLAG-tagged cyclin E gene constructs EL and LMW-E(T1) were cloned into the pcDNA 4.0 vector (Clontech, Mountain View, CA) and transfected into 76NE6 hMECs. Transfected cells were selected with 80 µg/mL zeocin (Invitrogen), and stable transfectants were maintained in culture with 10 µg/mL zeocin.

To generate 76NE6 cells with tetracycline-inducible cyclin E expression we used a similar strategy to the one we used for generation of the inducible cyclin E expression in MCF-7 cells [27]. Specifically, the cyclin E gene constructs were cloned into the pRetro-CMV/TO vector and transfected into 293T cells to produce retroviruses carrying the cyclin E constructs, and the pBMN-BSR-TetR vector to produce retroviruses carrying the Tet repressor.The 76NE6 cells were infected first with the retroviruses carrying the Tet repressor (TetR) gene fused with blasticidin-S resistance gene and then with the retroviruses carrying the cyclin E constructs. 76NE6 cells inducibly expressing TetR-vector, EL, LMW-E(T1), EL^R130A^, and LMW-E(T1^R130A^), were maintained in DFCI-1 medium with 20 µg/ml blasticidin-S and 1 µg/ml puromycin (InvivoGen, San Diego, CA). Other cell lines used in this study (i.e. Hs578T and MDA-MB-231) were obtained from American Type Tissue Collection and cultured as described previously [54].

### Tumorigenic assay and *in vivo* passaging

Nude mice were purchased from Charles River Laboratories (Wilmington, MA) and maintained in the Department of Veterinary Medicine at The University of Texas MD Anderson Cancer Center. The mice were injected subcutaneously in the mammary fat pad with 1×10^6^ cells suspended in 100 µL of a 1∶1 Matrigel∶media mix (Matrigel from BD Biosciences, San Diego, CA). Doxycycline was added to drinking water containing 1% sucrose, and water was replaced twice weekly. Mice were sacrificed under an Animal Care and Use Committee (ACUF)-approved protocol when tumors reached approximately 12 mm in diameter or 10 weeks after injection, whichever came first. The tumors were harvested for histopathological analysis or for expansion of tumor cells in tissue culture for reinjection into mice for *in vivo* passaging. Tumors submitted for histopathology were fixed in 10% neutral buffered formalin, paraffin embedded, and serially sectioned at 5 µm thickness.

### Reverse transcriptase PCR and real-time (RT–PCR)

RNA was isolated from mammary glands and tumors by using the RNAeasy kit (Qiagen). Reverse transcription (RT) was performed using the First Strand cDNA Synthesis Kit using 1 µg of mRNA per reaction (Roche). Real-time PCR was performed on the reverse-transcribed samples using SYBR Green PCR Master Mix (Applied Biosystems). RT reactions in which no reverse transcriptase had been added served as a monitor for the efficiency of the DNase I digestion. All reactions were carried out in triplicate. The fold difference in mouse cyclin E transcripts was calculated by the ΔΔCT method using GAPDH as the internal control. For every reaction, we observed a single peak on the dissociation curve plot. Primer sequences were as follows: cyclin E-F, 5′-CAGAGCAGCGAGCAGGAGA-3′; cyclin E-R, 5′ CAGCTGCTTCCACACCACTG-3′; GAPDH-F, 5′-TGTACCGTCTAGCATATCTCCGAC-3′; GAPDH-R, 5′-ATGATGTGCTCTAGCTCTGGGTG-3′.

### Transgenic mice

Transgenic mice with conditional expression of LMW-E were generated using the tetracycline regulatory system by cloning the coding sequence of LMW-E downstream of the minimal Tet operator in TMILA plasmid (a gift from L. A. Chodosh, University of Pennsylvania, Philadelphia, PA). Additionally, an IRES-firefly luciferase expression cassette was cloned downstream of LMW-E to serve as a surrogate reporter for transgene expression. Two founder mice (line 4372 and line 4382) harboring this *TetO-LMW-E* transgene (referred to as TLMW) were mated to a transgenic mouse harboring the MMTV-rtTA-pA transgene (referred to as MTB) to yield bi-transgenic MTB/TLMW mice [55]. Bi-transgenic mice carrying both of these transgenes express the rtTA transactivator in the mammary epithelium but do not express LMW-E unless doxycycline is added. TLMW founder line was generated by pronuclear injection and crossed with MTB mice. Whole-mount and bromodeoxyuridine (BrdU) staining were done as previously described [32].

### Staining of tumor sections

Paraffin-embedded tumor sections were stained with hematoxylin and eosin and for cyclin E using a polyclonal anti-cyclin E antibody (Santa Cruz Biotechnology, Santa Cruz, CA). The immunostaining was done as previously described [32] using a Vectastain ABC kit (per manufacturer's web site) and a BCIP/NBT chromogen detection system (Vector Laboratories, Burlingame, CA). Briefly, the sections were incubated in 1% H_2_O_2_ to block endogenous peroxidase activity and then incubated for 20 min in 10 mmol/L sodium citrate buffer (pH 6.0) at 90°C to retrieve nuclear antigens. Both primary and secondary antibody incubations were performed for 1 hr in blocking buffer (5% bovine serum albumin and 0.5% Tween-20 in 1× phosphate-buffered saline [PBS]) at room temperature. Nuclei were counterstained with hematoxylin.

### Transwell invasion assay

For each sample, 100 µl of 1 mg/ml Matrigel in serum free-cold MEM media was aliquoted into the upper chamber of 24-well transwell plate (Corning, Corning, NY) and incubated at 37°C for at least 4–5 hours for adequate gelling. The cells were washed and suspended in serum free medium at a 1×10^6^ cells/ml concentration. One hundred µl of cell suspension was transferred onto the upper chamber containing the Matrigel layer. The lower chamber of the transwell was filled with 600 µl of complete media containing 10 µg/ml fibronectin as an adhesive substrate. After 24 hours, the cells were fixed with 4% formaldehyde for 15 minutes, rinsed with PBS, and stained with 0.2% crystal violet for 10 minutes. The crystal violet was rinsed with excess ddH_2_O and the top chamber containing the Matrigel was thoroughly cleansed with Q-tips and the invaded cells were photographed with a light microscope. For quantification, the cells on the top and bottom of the chamber are collected using trypsin and counted using the culture counter. Each sample was counted 3 times and each experiment was repeated independently 3 times.

### Morphogenesis assay and drug treatment

3D culture on basement membrane was performed as described previously [21]. Assay medium (DFCI-1 medium with 2% growth factor-reduced Matrigel) with or without drugs was replaced every 4 days, and cells were cultured for 15 days.

### Indirect immunofluorescence analysis

Indirect immunofluorescence analysis of 3D cultures was performed as previously described with minor modifications [56]. Cells cultured in 8-well chamber slides were fixed with 2% paraformaldehyde at room temperature for 20 min, permeabilized with 1% Triton X-100 in PBS for 20 min, washed thrice with PBS/glycine buffer (130 mM NaCl, 7 mM Na_2_HPO_4_, 3.5 mM NaH_2_PO_4_, and 100 mM glycine), and blocked with IF buffer (130 mM NaCl, 7 mM Na_2_HPO_4_, 3.5 mM NaH_2_PO_4_, 7.7 mM NaN_3_, 0.1% bovine serum albumin, 0.2% Triton X-100, and 0.05% Tween-20) plus 10% goat serum for 1 hr at room temperature. Primary antibodies (laminin V and α6-integrin [Chemicon, Billerica, MA], GM-130 [BD Biosciences], E-cadherin [BD Biosciences], and Ki67 [Abcam, Cambridge, MA]) were incubated in IF buffer at 1∶200 dilution overnight at 4°C. The cells were incubated with Alexa fluor-conjugated rabbit (488), mouse (594), or rat (680) secondary antibodies (Molecular Probes, Carlsbad, CA), counterstained with 4,6-diamidino-2-phenylindole (DAPI) (Sigma, St. Louis, MO) for 15 min at room temperature, and mounted with antifade solution (Molecular Probes). Confocal microscopy was performed at room temperature using an Olympus FV300 laser scanning confocal microscope (Olympus America, Inc., Center Valley, PA) at 40× magnification, and images were processed using Adobe Photoshop (Version 11.0.2). For quantification, the diameter of each acinus was measured, and unpaired Student *t* test was used for statistical analysis.

### Protein isolation for Western blot and kinase assay

Cell lysates were prepared and subjected to Western blot analysis as described previously [12]. To obtain lysates from acini, acini were washed once with cold PBS, scraped, collected, and washed twice with cold PBS. Cell recovery solution (BD Biosciences) was added to the Matrigel/acini mixture at 1∶1 volume, and cells were incubated on ice for 1 hr, washed with PBS, and lysed as described previously [13] The protein blots were incubated with primary antibodies (cyclin E, vinculin [Santa Cruz], β-actin [Chemicon], BIM [StressGen Biotechnologies, Victoria, British Columbia], FAK, b-Raf, ERK1/2, pERK1/2 (T202/Y204), pMEK1/2 (S217/221), pS6 (S235/236), mTOR, eIF4E, Akt, pAkt(T308), and pRb(S807/811) [Cell Signaling Technology, Danvers, MA]) at 4°C with gentle shaking overnight.

Kinase assay with histone H1 and GST-Rb as cyclin E substrates was performed as described previously [13].

### Reverse-phase protein array (RPPA) analysis

The RPPA approach was performed as previously described [34]. Cellular proteins that were prepared as described for western blotting were denatured by boiling in 1% SDS (with beta-mercaptoethanol) and diluted in five 2-fold serial dilutions in dilution buffer (lysis buffer containing 1% SDS). Serial diluted lysates were arrayed on nitrocellulose-coated slides (Grace Biolab) using Aushon 2470 Arrayer (Aushon BioSystems). Each sample was robotically printed in 5 fold serial dilutions on multiple slides including positive and negative controls prepared from mixed cell lysates or dilution buffer, respectively, as well as multiple cell lines incubated with and without growth factors to provide dynamic range.

Each slide was probed with a validated primary antibody plus a biotin-conjugated secondary antibody. Antibody targets were selected as being relevant to breast cancer through a literature review. Antibodies where then obtained and validated against each potential target. Only antibodies with a Pearson correlation coefficient between RPPA and western blotting of greater than 0.7 were used in reverse phase protein array study. Antibodies with a single or dominant band on western blotting were further assessed by direct comparison to RPPA using cell lines with differential protein expression or modulated with ligands/inhibitors or siRNA for phospho- or structural proteins, respectively.

The signal obtained was amplified using a Dako Cytomation-catalyzed system (Dako) and visualized by DAB colorimetric reaction. The slides were scanned, analyzed, and quantified using a customerized-software Microvigene (VigeneTech Inc.) to generate spot intensity.

Each dilution curve was fitted with a logistic model (“Supercurve Fitting” developed by the Department of Bioinformatics and Computational Biology in MD Anderson Cancer Center, “http://bioinformatics.mdanderson.org/OOMPA”). This fits a single curve using all the samples (i.e., dilution series) on a slide with the signal intensity as the response variable and the dilution steps are independent variable. The fitted curve is plotted with the signal intensities – both observed and fitted - on the y-axis and the log2-concentration of proteins on the x-axis for diagnostic purposes. The protein concentrations of each set of slides were then normalized by median polish, which was corrected across samples by the linear expression values using the median expression levels of all antibody experiments to calculate a loading correction factor for each sample.

### Breast cancer patient samples and clinical data

The clinical data from 267 breast cancer patients used in this study were reported previously [16]. Each patient had received a diagnosis of breast cancer between 1990 and 1995 at 1 of 12 hospitals in the Chicago area. The study was approved by the institutional review board of the Wadsworth Center (Albany, NY). In the study reported herein, we used tissue samples from a portion of these for RPPA analysis to investigate large-scale protein expression pattern.

### Statistical analysis

Each cell culture experiment was performed at least three times. Continuous outcomes were summarized with means and standard deviations. Comparisons among groups were analyzed by two-sided *t* test and Wilcoxon rank-sum test. These analyses were performed using SPSS software, version 12.0. The differences in tumor incidence between groups (for Table 1 and Table 2) were compared using Fisher's exact test. The analyses were performed using SAS (version 9.2).

For analysis of the RPPA data, the SuperCurve quantified concentration of the proteins were natural log transformed. The differences in protein expression levels between high- and low LMW-E or EL expressed patient groups were assessed using Wilcoxon Rank Sum test. For the hierarchical clustering of the 71 proteins common between the 2D culture, 3D culture and patient data sets, the data for each protein in each data set were standardized (subtraction of mean then divided by standard deviation) separately, and then the standardized data were combined. The combined data set contains 93 cell line samples from 2D and 3D each, with three technical replicates for each of the 31 unique samples, and 276 breast tumor patient samples.

Disease-specific survival (DSS) was calculated from the date of surgical resection of the primary tumor to the date of death or last follow-up. Data for patients who died from causes other than breast cancer were censored at the time of death. DSS curves were computed by the Kaplan-Meier method [57]. Bivariate analyses of DSS in patients with high LMW-E expression according to levels of FAK, BIM, total Akt, and pAkt(T308) were performed with the use of a two-sided log-rank test [58]. Kaplan-Meier survival curves were calculated for the different cyclin E groups, and the log-rank test was used to compare the disease-free survival among groups. Stata statistical software (SE 9, StataCorp LP, College Station, TX) was used for these statistical analyses. All *P* values were 2 tailed, and *p*<0.05 was considered significant.

## Supporting Information

Figure S1 LMW-E overexpression causes elevation of cyclin E protein and mRNA levels. (A) Cells were grown on Matrigel for 15 days and RNA were extracted and subjected to qRT-PCR analysis for the mRNA level of cyclin E and normalized against GAPDH mRNA levels. Statistical analysis used was unpaired student's *t*-test. (B) Mouse GAPDH and mouse cyclin E mRNA expression levels in tumor and contralateral mammary gland of 3 different LMW-E overexpressing transgenic mice. Quantitative real-time PCR (qRT–PCR) was performed on a 7300 Real-Time PCR System from Applied Biosystems.
(PPT)

Figure S2 Hierarchichal clustering of patient samples and proteins using the top 50 differentially expressed proteins between high and low LMW-E or EL. Proteins were ranked by the p-values based on two-sample t-test on the natural log transformed concentration. Row side color indicates cyclin E expression. (Red: samples with low cyclin E expression; blue: samples with high cyclin E expression.) (A) Use the top 50 differentially expressed proteins between high and low EL. (B) Use the top 50 differentially expressed proteins between high and low LMW-E.
(PPT)

Figure S3 Box plots of protein expression in patient tumors by low versus high cyclin E levels. Differences in the expression of these proteins between samples with high and low LMW-E or between high and low EL cyclin E were compared using the Wilcoxon Rank-Sum tests.
(PPT)

Figure S4 Combination drug treatment prevents induction of aberrant acinar development by LMW-E. Cells were seeded on Matrigel for 24 hours and then treated as indicated. Medium containing drugs was replaced every 4 days. On day 15 of Matrigel culture, cells were fixed and stained with E-cadherin (red) and Ki67 (green), and nuclei were counterstained with DAPI (blue). Scale bar = 50 µm. (B) The diameters of the acini were measured and averaged from three independent experiments. Error bars = SEM (Student *t* test, *p<0.05). (C) The number of Ki67-positive cells per acinus was counted and averaged from three independent experiments. Error bars = SEM (Student *t* test, *p<0.05).
(PPT)

Figure S5 Combination drug treatment prevents induction of aberrant acinar development by LMW-E. (Quantitation of the study from Figure S4) Cells were seeded on Matrigel for 24 hours and then treated as indicated. Medium containing drugs was replaced every 4 days. (A) On day 15 of Matrigel culture, the diameters of the acini were measured and averaged from three independent experiments. Error bars = SEM (Student *t* test, *p<0.05). (B) The number of Ki67-positive cells per acinus was counted and averaged from three independent experiments. Error bars = SEM (Student *t* test, *p<0.05).
(PPT)

Figure S6 Activated b-Raf-ERK1/2-mTOR signaling pathway and high EL expression does not predict poor survival in breast cancer patients. Kaplan-Meier survival plots demonstrating association between full length and high LMW-E in association with FAK, BIM, Akt and pAkt (T308) protein levels obtained from RPPA analysis.
(PPT)

Figure S7 High EL expression does not associate with the b-Raf-ERK1/2-mTOR pathway to predict patient DSS. Kaplan-Meier survival plots demonstrating association between combination of FAK, BIM, Akt and pAkt (T308) protein levels on disease-specific survival in patients with high full length. The expression levels of FAK, BIM, Akt and pAkt (T308) were dichotomized using their median values from all 276 patient samples. All p values are based on log-rank test.
(PPT)

Table S1 List of proteins used in the RPPA assay.
(DOC)

Table S2 Proteins that show distinct expression pattern between 2D and 3D cultures.
(DOC)

Table S3 Disease-specific survival rates of proteins that associate with high LMW-E protein levels.
(PPT)

Table S4 Statistical analysis of the effects of the combination drug treatment on acinar diameter.
(PPT)

Table S5 Statistical analysis of the effects of the combination drug treatment on the percentage of Ki67-positive acini.
(PPT)
