# Supplementary material for: LMW-E/CDK2 Deregulates Acinar Morphogenesis, Induces Tumorigenesis, and Associates with the Activated b-Raf-ERK1/2-mTOR Pathway in Breast Cancer Patients
Source: PLoS Genet. 2012 Mar 29;8(3):e1002538. doi: 10.1371/journal.pgen.1002538 (PMC3315462; doi:10.1371/journal.pgen.1002538)
Supplement: Figure S1 — LMW-E overexpression causes elevation of cyclin E protein and mRNA levels. (A) Cells were grown on Matrigel for 15 days and RNA were extracted and subjected to qRT-PCR analysis for the mRNA level of cyclin E and normalized against GAPDH mRNA levels. Statistical analysis used was unpaired student's t-test. (B) Mouse GAPDH and mouse cyclin E mRNA expression levels in tumor and contralateral mammary gland of 3 different LMW-E overexpressing transgenic mice. Quantitative real-time PCR (qRT–PCR) was performed on a 7300 Real-Time PCR System from Applied Biosystems. (PPT) [file pgen.1002538.s001.ppt]

## Slide 1
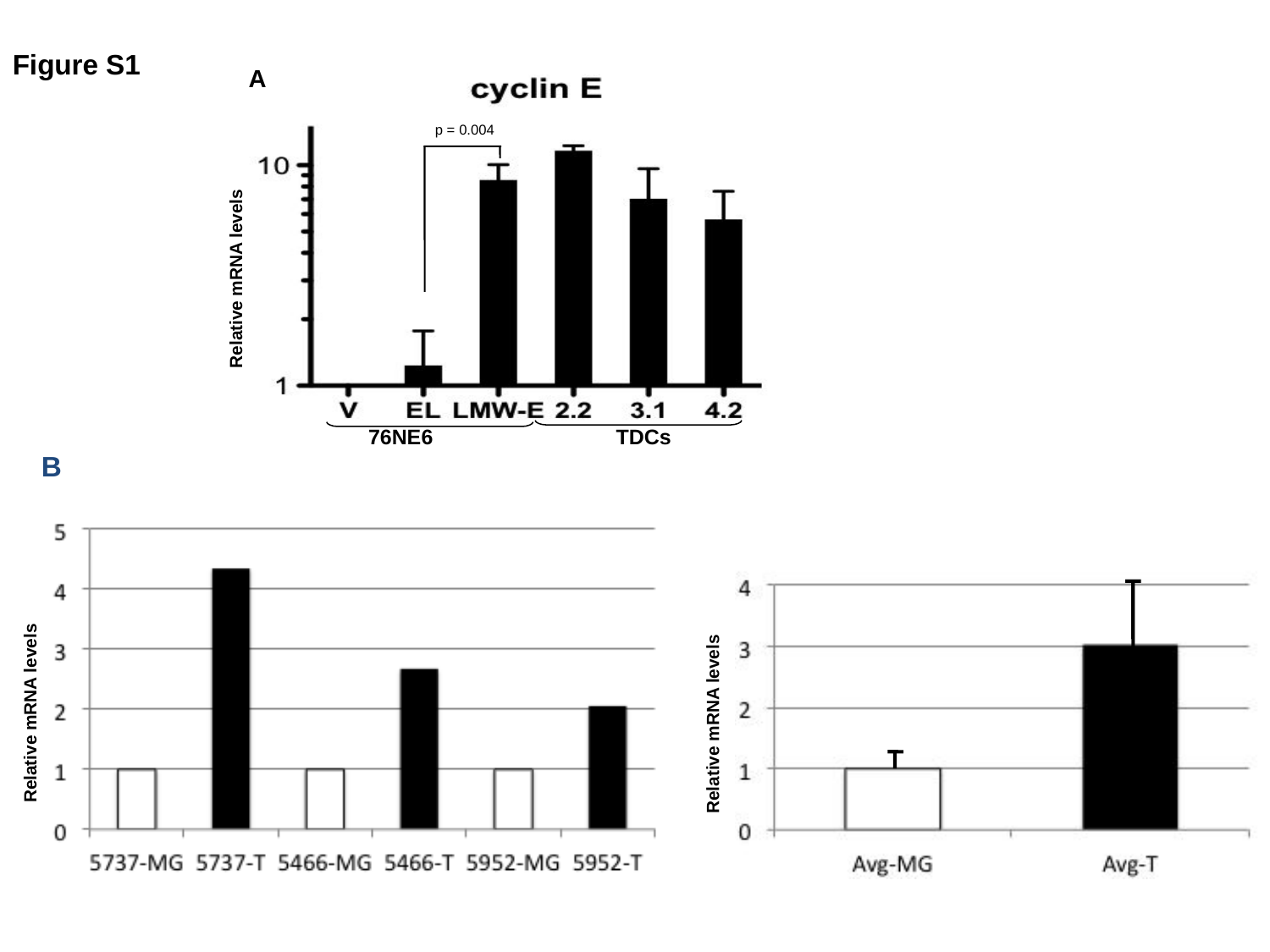

p = 0.004
76NE6 TDCs
Figure S1
A
Relative mRNA levels
 B
Relative mRNA levels
Relative mRNA levels
Figure 19: LMW-E activates gene expression associated with the EMT. (A – F) Cells were grown in monolayer culture to 70% confluency and RNA were extracted and subjected to qRT-PCR analysis for the mRNA levels of cyclin E (A), E-cadherin (B), twist (C), vimentin (D), N-cadherin (E), and slug (F). These values were normalized against GAPDH mRNA levels and statistical analysis used was unpaired student’s t-test.
Figure 19: LMW-E activates gene expression associated with the EMT. (A – F) Cells were grown in monolayer culture to 70% confluency and RNA were extracted and subjected to qRT-PCR analysis for the mRNA levels of cyclin E (A), E-cadherin (B), twist (C), vimentin (D), N-cadherin (E), and slug (F). These values were normalized against GAPDH mRNA levels and statistical analysis used was unpaired student’s t-test.
Figure 19: LMW-E activates gene expression associated with the EMT. (A – F) Cells were grown in monolayer culture to 70% confluency and RNA were extracted and subjected to qRT-PCR analysis for the mRNA levels of cyclin E (A), E-cadherin (B), twist (C), vimentin (D), N-cadherin (E), and slug (F). These values were normalized against GAPDH mRNA levels and statistical analysis used was unpaired student’s t-test.
