# Supplementary figures and images for: LMW-E/CDK2 Deregulates Acinar Morphogenesis, Induces Tumorigenesis, and Associates with the Activated b-Raf-ERK1/2-mTOR Pathway in Breast Cancer Patients
Source: PLoS Genet. 2012 Mar 29;8(3):e1002538. doi: 10.1371/journal.pgen.1002538 (PMC3315462; doi:10.1371/journal.pgen.1002538)

## Slide 1
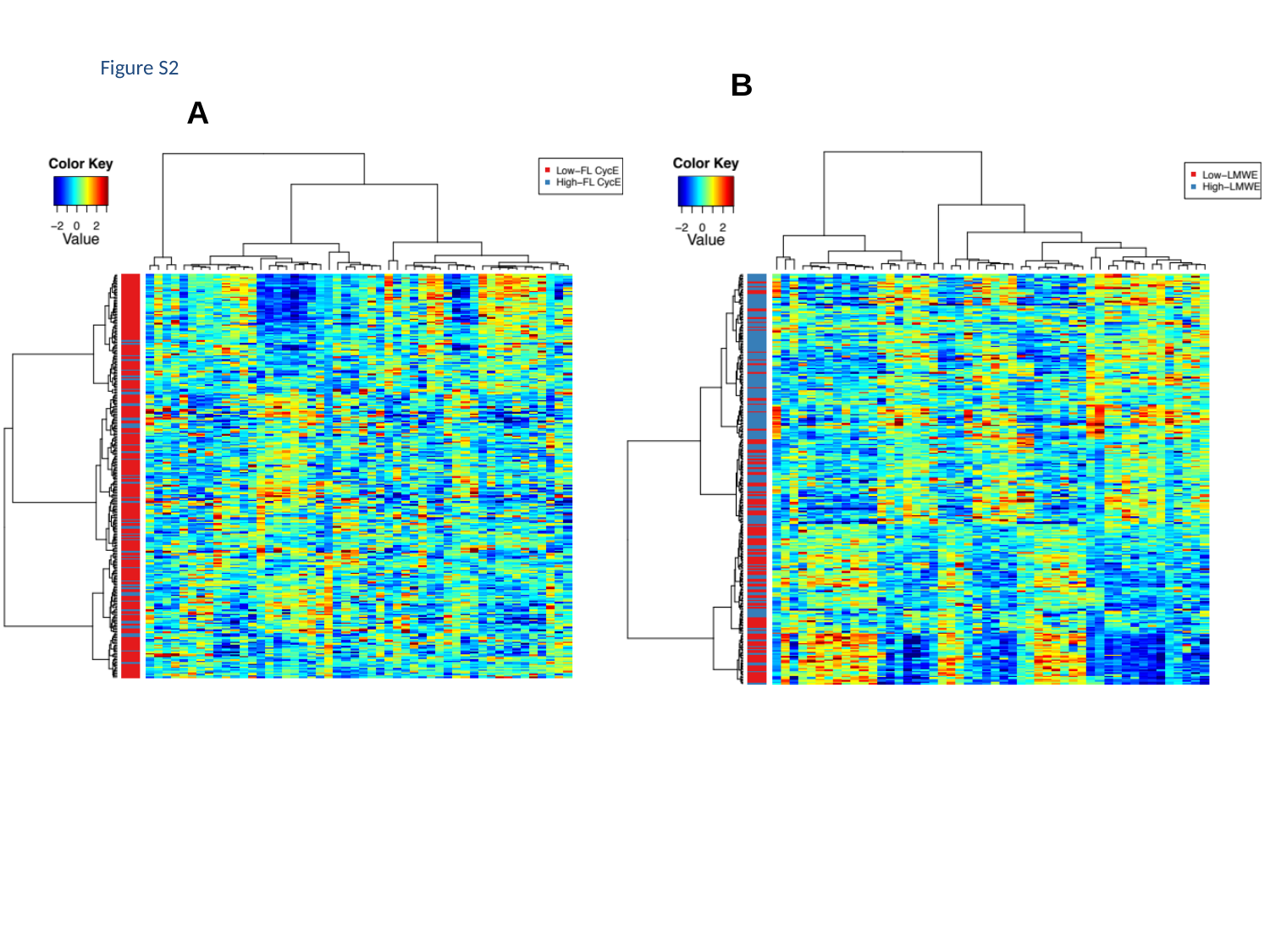

Figure S2
B
A

Supplement: Figure S2 — Hierarchichal clustering of patient samples and proteins using the top 50 differentially expressed proteins between high and low LMW-E or EL. Proteins were ranked by the p-values based on two-sample t-test on the natural log transformed concentration. Row side color indicates cyclin E expression. (Red: samples with low cyclin E expression; blue: samples with high cyclin E expression.) (A) Use the top 50 differentially expressed proteins between high and low EL. (B) Use the top 50 differentially expressed proteins between high and low LMW-E. (PPT) [file pgen.1002538.s002.ppt]

## Slide 1
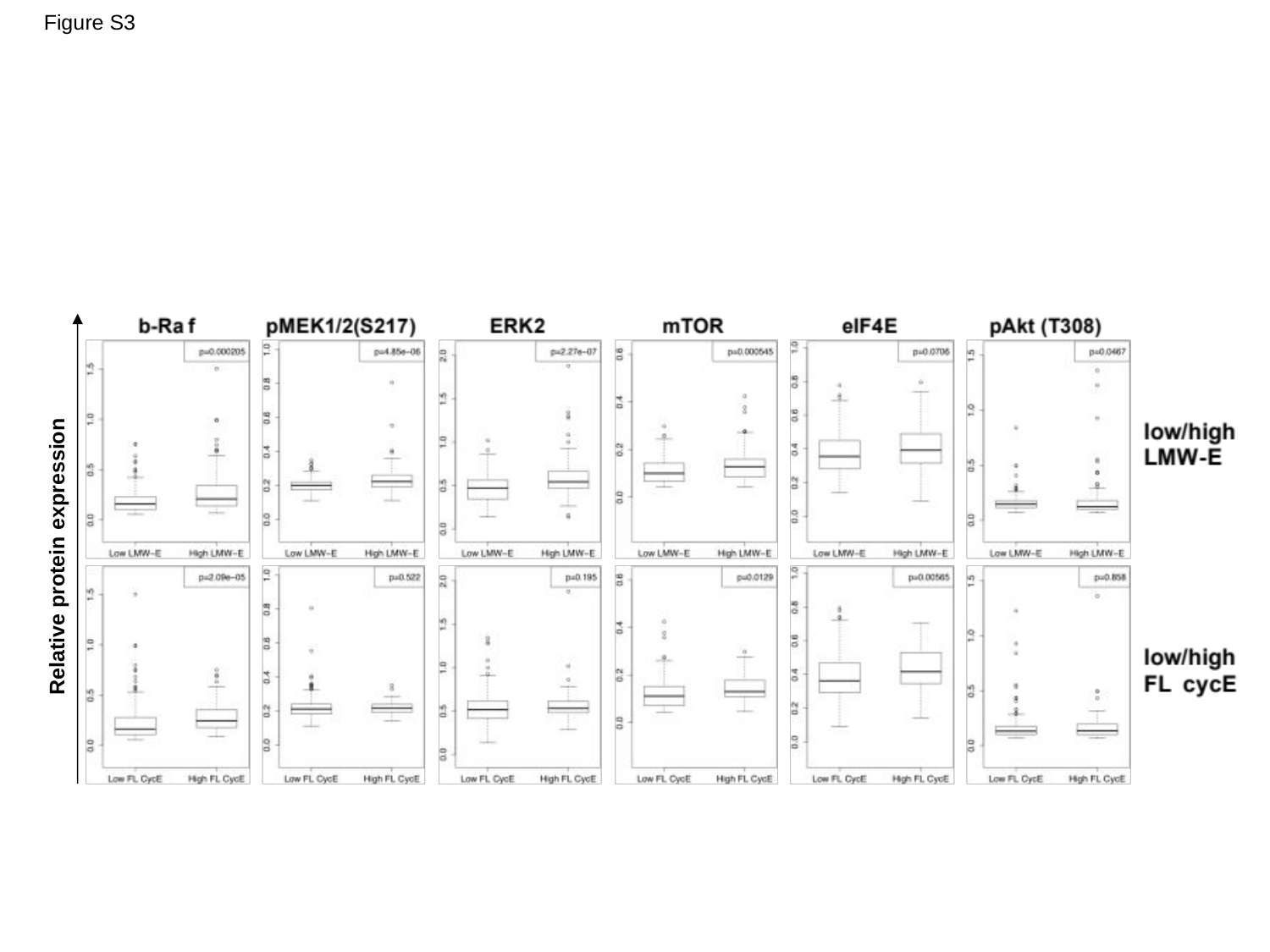

# Figure S3
Relative protein expression

Supplement: Figure S3 — Box plots of protein expression in patient tumors by low versus high cyclin E levels. Differences in the expression of these proteins between samples with high and low LMW-E or between high and low EL cyclin E were compared using the Wilcoxon Rank-Sum tests. (PPT) [file pgen.1002538.s003.ppt]

## Slide 1
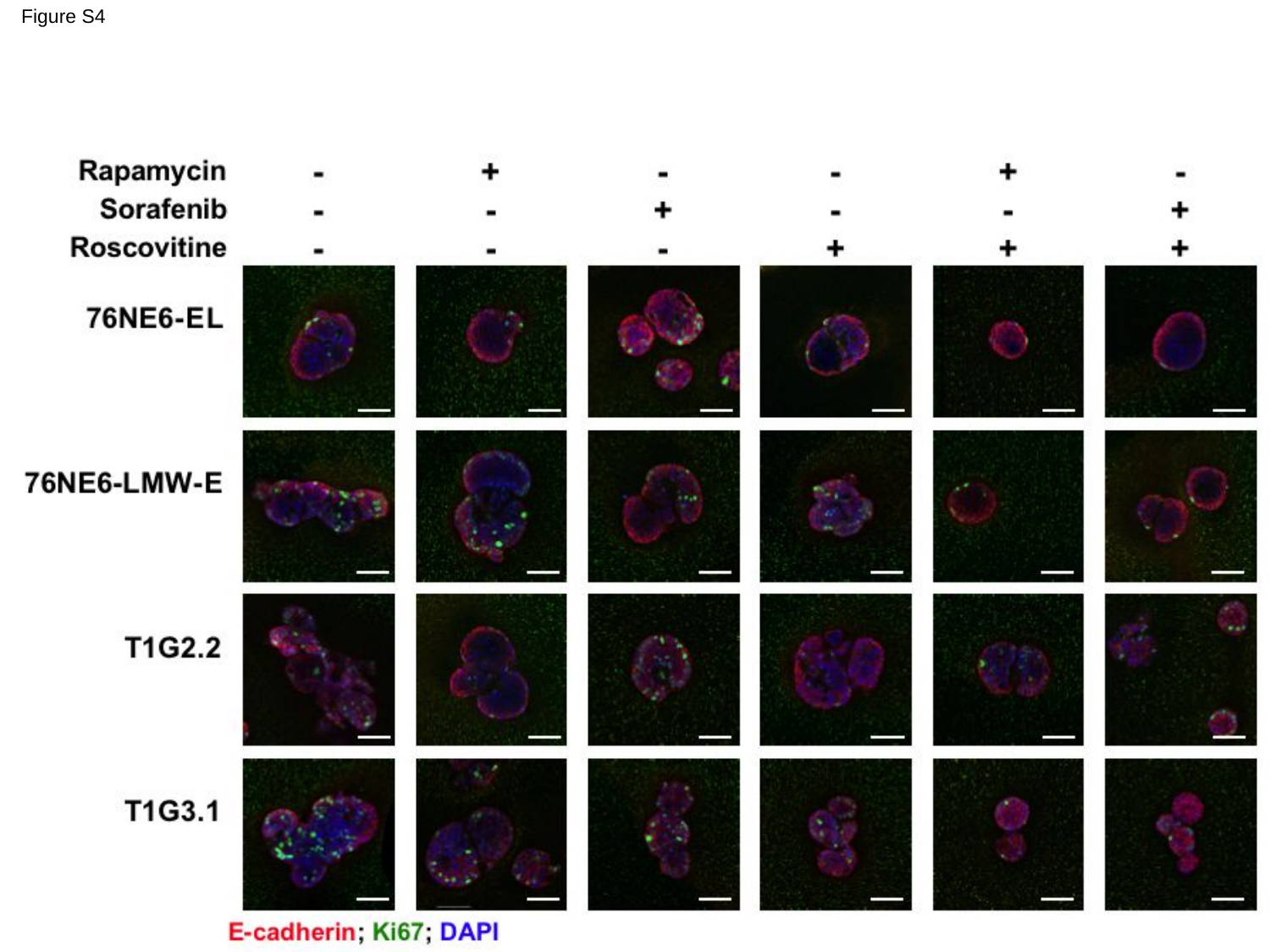

# Figure S4

Supplement: Figure S4 — Combination drug treatment prevents induction of aberrant acinar development by LMW-E. Cells were seeded on Matrigel for 24 hours and then treated as indicated. Medium containing drugs was replaced every 4 days. On day 15 of Matrigel culture, cells were fixed and stained with E-cadherin (red) and Ki67 (green), and nuclei were counterstained with DAPI (blue). Scale bar = 50 µm. (B) The diameters of the acini were measured and averaged from three independent experiments. Error bars = SEM (Student t test, *p<0.05). (C) The number of Ki67-positive cells per acinus was counted and averaged from three independent experiments. Error bars = SEM (Student t test, *p<0.05). (PPT) [file pgen.1002538.s004.ppt]

## Slide 1
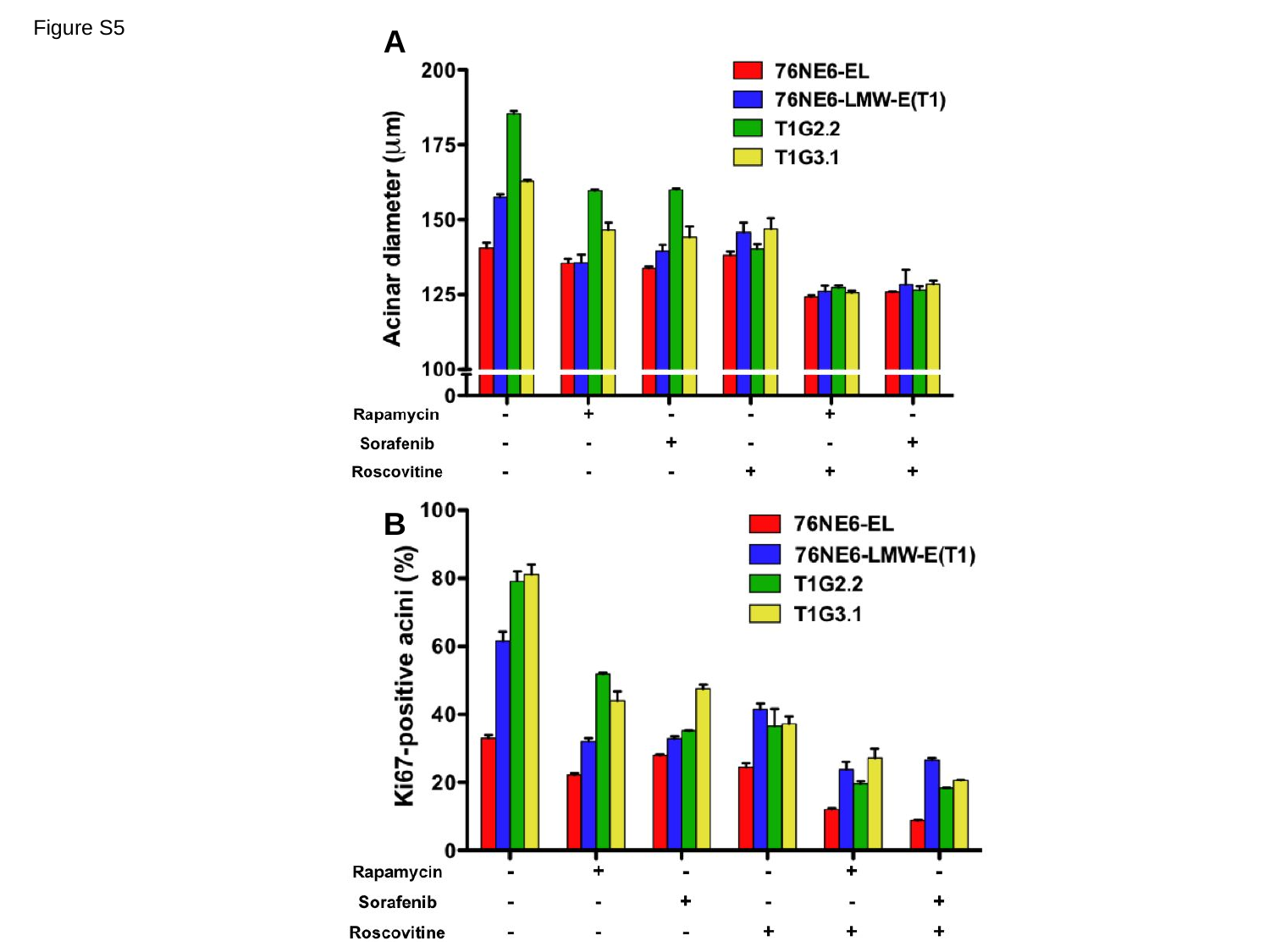

# Figure S5
A
B

Supplement: Figure S5 — Combination drug treatment prevents induction of aberrant acinar development by LMW-E. (Quantitation of the study from Figure S4) Cells were seeded on Matrigel for 24 hours and then treated as indicated. Medium containing drugs was replaced every 4 days. (A) On day 15 of Matrigel culture, the diameters of the acini were measured and averaged from three independent experiments. Error bars = SEM (Student t test, *p<0.05). (B) The number of Ki67-positive cells per acinus was counted and averaged from three independent experiments. Error bars = SEM (Student t test, *p<0.05). (PPT) [file pgen.1002538.s005.ppt]

## Slide 1
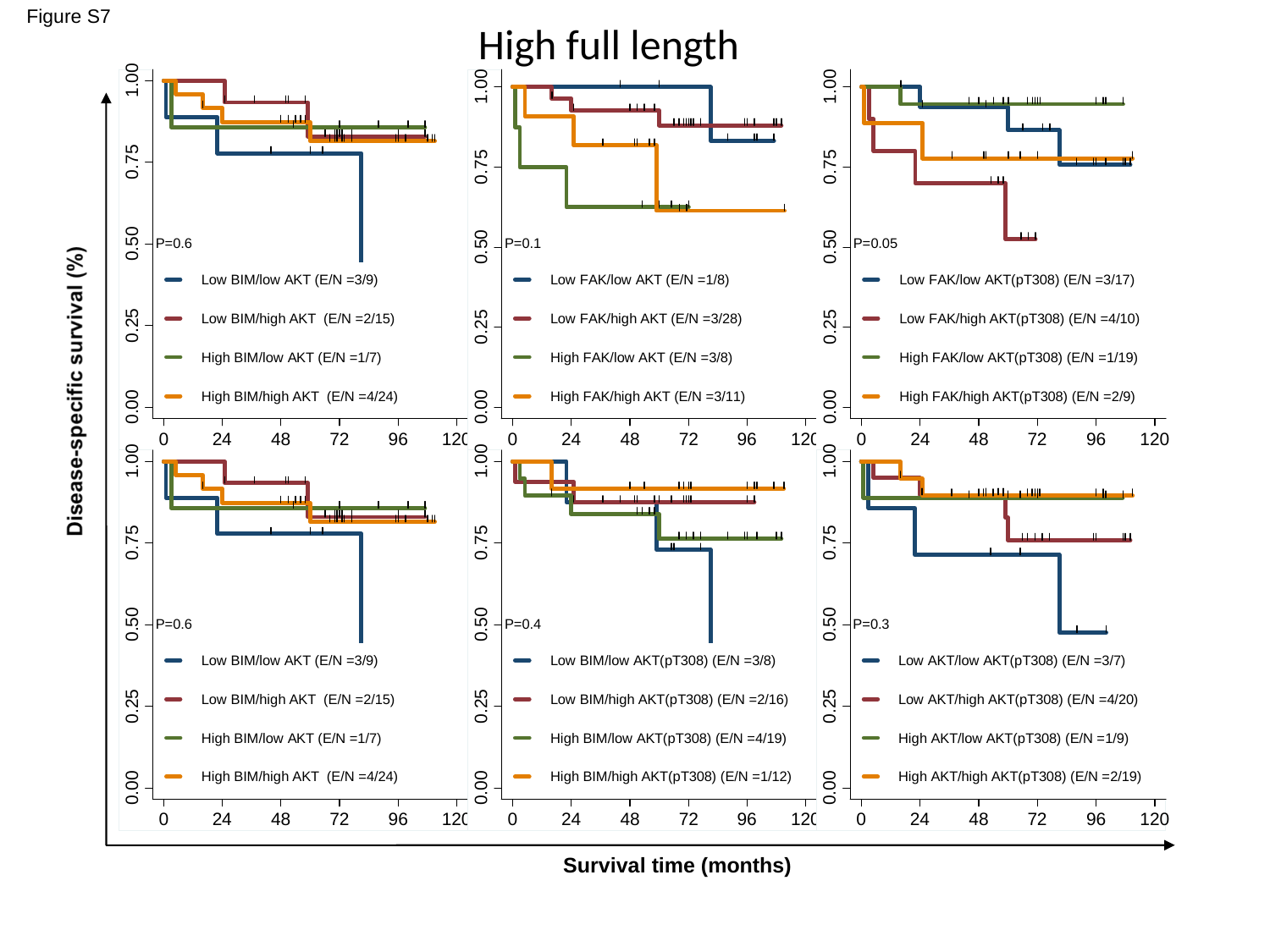

# Figure S7
High full length
Survival time (months)

Supplement: Figure S7 — High EL expression does not associate with the b-Raf-ERK1/2-mTOR pathway to predict patient DSS. Kaplan-Meier survival plots demonstrating association between combination of FAK, BIM, Akt and pAkt (T308) protein levels on disease-specific survival in patients with high full length. The expression levels of FAK, BIM, Akt and pAkt (T308) were dichotomized using their median values from all 276 patient samples. All p values are based on log-rank test. (PPT) [file pgen.1002538.s007.ppt]
