# Supplementary material for: LMW-E/CDK2 Deregulates Acinar Morphogenesis, Induces Tumorigenesis, and Associates with the Activated b-Raf-ERK1/2-mTOR Pathway in Breast Cancer Patients
Source: PLoS Genet. 2012 Mar 29;8(3):e1002538. doi: 10.1371/journal.pgen.1002538 (PMC3315462; doi:10.1371/journal.pgen.1002538)
Supplement: Figure S6 — Activated b-Raf-ERK1/2-mTOR signaling pathway and high EL expression does not predict poor survival in breast cancer patients. Kaplan-Meier survival plots demonstrating association between full length and high LMW-E in association with FAK, BIM, Akt and pAkt (T308) protein levels obtained from RPPA analysis. (PPT) [file pgen.1002538.s006.ppt]

## Slide 1
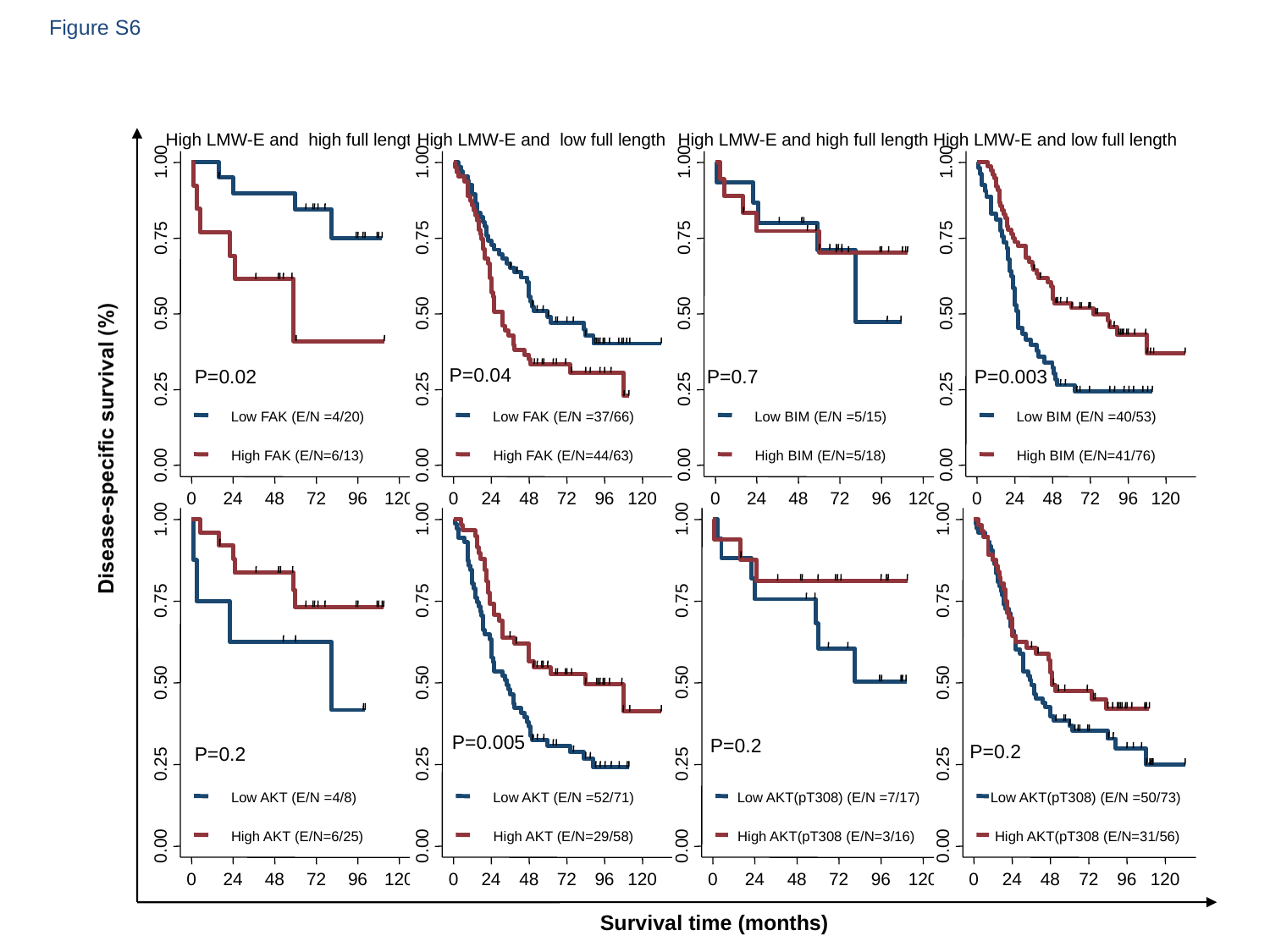

Figure S6
High LMW-E and high full length
High LMW-E and low full length
High LMW-E and high full length
1.00
1.00
1.00
0.75
0.75
0.75
0.50
0.50
0.50
P=0.04
P=0.02
P=0.7
0.25
0.25
0.25
Low FAK (E/N =4/20)
Low FAK (E/N =37/66)
Low BIM (E/N =5/15)
High FAK (E/N=6/13)
High FAK (E/N=44/63)
High BIM (E/N=5/18)
0.00
0.00
0.00
0
24
48
72
96
120
0
24
48
72
96
120
0
24
48
72
96
120
High LMW-E and low full length
1.00
0.75
0.50
P=0.003
0.25
Low BIM (E/N =40/53)
High BIM (E/N=41/76)
0.00
0
24
48
72
96
120
1.00
1.00
0.75
0.75
0.50
0.50
P=0.005
P=0.2
0.25
0.25
Low AKT (E/N =4/8)
Low AKT (E/N =52/71)
High AKT (E/N=6/25)
High AKT (E/N=29/58)
0.00
0.00
0
24
48
72
96
120
0
24
48
72
96
120
1.00
1.00
0.75
0.75
0.50
0.50
P=0.2
P=0.2
0.25
0.25
Low AKT(pT308) (E/N =7/17)
Low AKT(pT308) (E/N =50/73)
High AKT(pT308 (E/N=3/16)
High AKT(pT308 (E/N=31/56)
0.00
0.00
0
24
48
72
96
120
0
24
48
72
96
120
Survival time (months)
