# Supplementary material for: LMW-E/CDK2 Deregulates Acinar Morphogenesis, Induces Tumorigenesis, and Associates with the Activated b-Raf-ERK1/2-mTOR Pathway in Breast Cancer Patients
Source: PLoS Genet. 2012 Mar 29;8(3):e1002538. doi: 10.1371/journal.pgen.1002538 (PMC3315462; doi:10.1371/journal.pgen.1002538)
Supplement: Table S1 — List of proteins used in the RPPA assay. (DOC) [file pgen.1002538.s008.doc]

| **Table S1. List of proteins used in the RPPA assay** | | | |
| --- | --- | --- | --- |
| The protein set used to analyze the patient samples contained a total of 125 proteins, of which 74 of the proteins overlap with the cell line sample analysis (red). | | | |
| 1. ACC pS79 | 1. MAPK pT202/204 | 1. aB crystallin | 1. HSP70 |
| 1. AIB1 | 1. mTOR | 1. ACC1 | 1. IGFRb |
| 1. AKT | 1. p21 | 1. annexin | 1. JNK pT183 |
| 1. Akt pS473 | 1. p27 | 1. ATM | 1. MEK1/2 pS217 |
| 1. AKT pS473 | 1. p38 pT180/Y182 | 1. ATR pS423 | 1. MME CD10 |
| 1. AKT pT308 | 1. p53 | 1. ATRIP | 1. MSH2 |
| 1. AMPK A pT172 | 1. p70 S6K | 1. b-Raf | 1. N-cadherin |
| 1. AMPK A | 1. p70 S6K pT389 | 1. BAD pS112 | 1. NFKBp65 pS536 |
| 1. AR | 1. 46. PDK1 pS241 | 1. BAX | 1. p70S6K pS371 |
| 1. beta-catenin | 1. 47. PKC-A | 1. Bcl-X | 1. PAI1 |
| 1. beta-catenin pS33 | 1. PKC-A pS657 | 1. Bcl-xL | 1. cleaved PARP |
| 1. Bcl2 | 1. PR | 1. Bcl2 | 1. cleaved PARP |
| 1. BIM | 1. PTEN | 1. BID | 1. PAX2 |
| 1. cKIT | 1. Rb | 1. c-Raf | 1. PCNA |
| 1. cMYC | 1. Rb pS807/811 | 1. CF | 1. PKCE pT40 |
| 1. Cleaved Capase 7 | 1. S6 | 1. CHK1 | 1. SMAD1 |
| 1. Caveolin | 1. S6 pS235/236 | 1. CHK2 | 1. SMAD4 |
| 1. CD31 | 1. S6 pS240/244 | 1. ER pS167 | 1. STAT6 pY641 |
| 1. COLLAGEN VI | 1. SMAD3 | 1. Fibronectin | 1. SYK |
| 1. Cyclin D1 | 1. SMAD3 pS432 | 1. Fortilin | 1. TAU |
| 1. CyclinE1 | 1. SRC | 1. FOXM1 | 1. Telomerase |
| 1. E-cadherin | 1. SRC pY527 | 1. FOXO3a | 1. XRCC1 |
| 1. EGFR | 1. STAT3 | 1. GATA3 | 1. YAP pY127 |
| 1. eIF4E | 1. STAT3 pT727 | 1. GSK3a/b | 1. YBI |
| 1. ELK-1_pS383 | 1. STAT3 pY705 | 1. GYS1 | 1. YBI pS112 |
| 1. ER pS118 | 1. STAT5 | 1. GYS1 pS640 |  |
| 1. ERK2 | 1. STAT5 pY694 |  |  |
| 1. ETV6 | 1. STAT6 pY641 |  |  |
| 1. FAK | 1. Stathmin |  |  |
| 1. FOXO3 A pS318/321 | 1. TSC2 |  |  |
| 1. GSK3 | 1. TSC2 pT1462 |  |  |
| 1. GSK3 pS21 | 1. VEGFR2 |  |  |
| 1. HER2 | 1. 14-3-3 Zeta |  |  |
| 1. HSP27 | 1. 4EBP1 |  |  |
| 1. IGFBP2 | 1. 4EBP1 pS65 |  |  |
| 1. IRS-1 | 1. 4EBP1 pT37/46 |  |  |
| 1. IRS-1 pS307 | 1. YAP |  |  |
