# Supplementary material for: LMW-E/CDK2 Deregulates Acinar Morphogenesis, Induces Tumorigenesis, and Associates with the Activated b-Raf-ERK1/2-mTOR Pathway in Breast Cancer Patients
Source: PLoS Genet. 2012 Mar 29;8(3):e1002538. doi: 10.1371/journal.pgen.1002538 (PMC3315462; doi:10.1371/journal.pgen.1002538)
Supplement: Table S2 — Proteins that show distinct expression pattern between 2D and 3D cultures. (DOC) [file pgen.1002538.s009.doc]

**Table S2**. Proteins that show distinct expression pattern between 2D and 3D cultures

| **Protein** | **Function(s)** |
| --- | --- |
| EGFR | transmembrane tyrosine kinase upstream of the MAPK, Akt, and JNK pathways regulating DNA synthesis and cell proliferation |
| PKC-a | upstream kinase that controls secretion, gene expression, proliferation and muscle contraction |
| ACC pS79 | catalyzes the pivotal step of the fatty acid synthesis pathway, phosphorylation by AMPK inhibits activity |
| AMPK A pT172 | regulates the metabolism of fatty acids and glycogen, controls protein synthesis and cell growth through E2F and TSC2/mTOR pathways |
| E-cadherin | mediates calcium-dependent cell-cell adhesion |
| Caveolin | regulates vesicular trafficking, cholesterol homeostasis, cell adhesion and apoptosis |
| B-catenin | downstream effector of Wnt signaling, unphosphorylated b-catenin activates gene transcription |
| GSK3 pS21 | regulates glycogen synthesis in response to insulin |
| PTEN | negative regulation of the PI3K/Akt pathway |
| Akt  Akt pS473 | promotes cell survival by inhibiting apoptosis |
| PDK1 pS241 | activates Akt, p70 S6K and RSK |
| mTOR | ATP and amino acid sensor to balance nutrient availability and cell growth |
| S6 pS235/236  S6 pS240/244 | controls translation of proteins involved in cell cycle progression, ribosomal proteins and elongation factors |
| p70S6K | regulates cell growth and G1 cell cycle progression |
| 4EBP1  4EBP1 pT37/46 | translation repressor protein, inhibits cap-dependent translation by binding to the translation initiation factor eIF4E  hyperphosphorylation of 4E-BP1 disrupts this interaction and results in activation of cap-dependent translation |
| e1F4E | cap-binding protein, rate-limiting factor for initiation of translation |
| STAT3 pS727  STAT3 | transcription factor downstream of cytokines and growth factor receptors |
| SMAD3 | regulates gene expression by transmitting TGF-β signals from the cell surface into the nucleus |
| AIB1 | (SRC-3) transcriptional co-regulator of nuclear receptors and other transcription factors |
| ETV6 | important for hematopoiesis and maintenance of the developing vascular network |
| YAP | binds to SH3 domains and play a role in anchoring and targeting to specific subcellular compartments |
| Rb pS807/811  Rb | control G1-S phase transition |
