# Supplementary material for: LMW-E/CDK2 Deregulates Acinar Morphogenesis, Induces Tumorigenesis, and Associates with the Activated b-Raf-ERK1/2-mTOR Pathway in Breast Cancer Patients
Source: PLoS Genet. 2012 Mar 29;8(3):e1002538. doi: 10.1371/journal.pgen.1002538 (PMC3315462; doi:10.1371/journal.pgen.1002538)
Supplement: Table S3 — Disease-specific survival rates of proteins that associate with high LMW-E protein levels. (PPT) [file pgen.1002538.s010.ppt]

## Slide 1
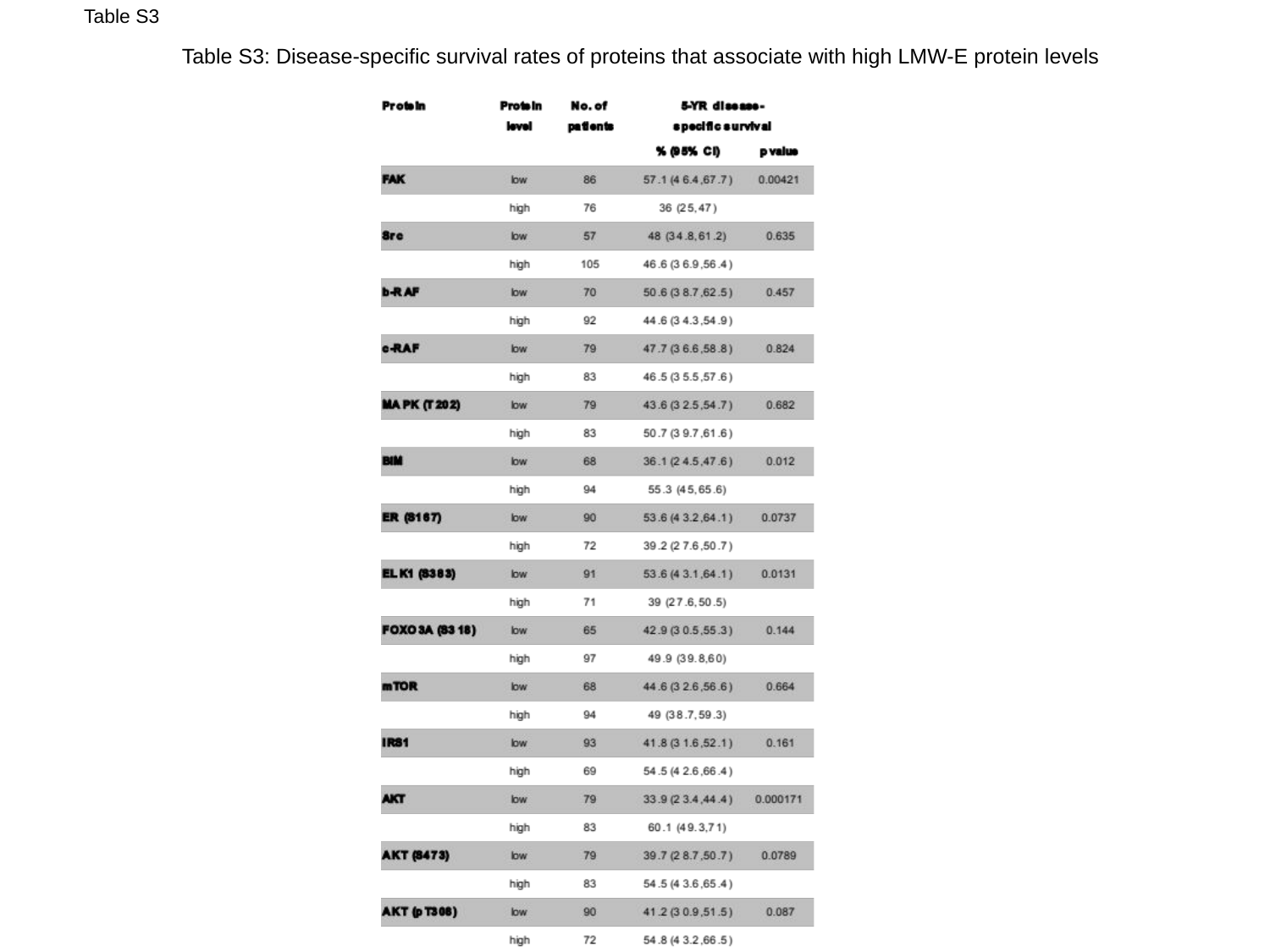

# Table S3
Table S3: Disease-specific survival rates of proteins that associate with high LMW-E protein levels
